# Large scale screening of CRISPR guide RNAs using an optimized high throughput robotics system

**DOI:** 10.1038/s41598-022-17474-8

**Published:** 2022-08-17

**Authors:** J. R. Spangler, T. A. Leski, Z. Schultzhaus, Z. Wang, D. A. Stenger

**Affiliations:** 1 Center for Bio/Molecular Science & Engineering (Code 6900), US Naval Research Laboratory, Washington, DC USA; 2 Centers for Disease Control and Prevention, Atlanta, GA USA

**Keywords:** Biological techniques, High-throughput screening, Target identification, Assay systems, Nucleic-acid therapeutics

## Abstract

All CRISPR/CAS systems utilize CRISPR guide RNAs (crRNAs), the design of which depend on the type of CAS protein, genetic target and the environment/matrix. While machine learning approaches have recently been developed to optimize some crRNA designs, candidate crRNAs must still be screened for efficacy under relevant conditions. Here, we demonstrate a high-throughput method to screen hundreds of candidate crRNAs for activation of Cas13a collateral RNA cleavage. Entire regions of a model gene transcript (*Y. pestis* lcrV gene) were tiled to produce overlapping crRNA sets. We tested for possible effects that included crRNA/target sequence, size and secondary structures, and the commercial source of DNA oligomers used to generate crRNAs. Detection of a 981 nt target RNA was initially successful with 271 out of 296 tested guide RNAs, and that was improved to 287 out of 296 (97%) after protocol optimizations. For this specific example, we determined that crRNA efficacy did not strongly depend on the target region or crRNA physical properties, but was dependent on the source of DNA oligomers used for RNA preparation. Our high-throughput methods for screening crRNAs has general applicability to the optimization of Cas12 and Cas13 guide RNA designs.

## Introduction

The field of nucleic acid detection has exploded in recent years following the discovery of Clustered Regularly Interspersed Short Palindromic Repeat (CRISPR)-Cas systems^1^. Improved methods of predicting amino acid structure and function have allowed investigators to continue identifying CRISPR-Cas systems from new organisms, therefore growing the field and increasing potential for biotechnological innovation. Some examples include applications in microbial engineering^2,3^, agricultural engineering^4,5^, novel analyte detection^6^, disease-mediation^7^, stem cell development^8^ and viral detection^9^. CRISPR-Cas systems generally fall into two classes based on the number of required Cas components for full function, wherein Class 2 (containing types II, V, and VI) consists of single protein systems that are most useful for biotechnological development due to their relative simplicity^10^. There is considerable functional variability within this class, including DNA-identifying Cas9 that is able to hydrolyze specific DNA sequences and initiate directed genome editing, along with other proteins that utilize the RNA-identifying Cas12 to cleave DNA reporters^9^ or Cas13 to cleave RNA reporters^11^ that have been used as highly sensitive methods for nucleic acid detection.

The recent global focus on genetic target detection has been met with the design of multiple systems to streamline the application of CRISPR-Cas to this end, and this topic has been the focus of extensive recent study^12–14^ and review^15,16^. One particular application of interest involves coupling target amplification with Cas13a RNase activity that offers reproducible single molecule detection of nucleic acids with striking sequence fidelity^11^. The sensitivity of this assay, termed SHERLOCK, has generated immense interest for its potential in many areas of diagnostics^9^, sparked the generation of multiple companies, and become the focus of research programs for its transition to field applications. Such interest has additionally brought attention to increasing the accessibility of CRISPR-based detection, as there is an apparent need for universal and simplified crispr RNA (crRNA) design rules. Many investigators have dedicated work towards studying the required RNA structure and protein interactions with various Cas systems^17,18^ (and reviewed in^19^), but a universal ruleset for guide RNA design seems to be fleeting. There are many examples of design strategies proven successful in a variety of settings as discussed above, however each crRNA or CRISPR-array design is dependent on its associated Cas protein, and each of these harbor slight variations in sequence fidelity and length intrinsic to their unique properties. Therefore, all CRISPR applications require some degree of optimization, prompting some investigators to develop algorithms^20^ and methods^21,22^ to relieve these bottlenecks in the interest of accessibility. Extensive trial and error, however, is still common for the understanding and optimization of many applications.

Recent investigations have shown the SHERLOCK assay to be a successful viral detection strategy^9^, and subsequent studies have demonstrated the application of these principles to differentially identify the *lcrV* gene from three *Yersinia* spp. with high sequence similarity^23^. This gene encodes a *Y. pestis* virulence factor that has evaded antigen-based detection methods. Furthermore, the investigators optimized shortcuts in the SHERLOCK protocol that greatly reduced bench time without sacrificing detection sensitivity to allow for rapid assessment of hundreds of tiled crRNAs. Tiled crRNAs in that study, however, yielded variable Cas13a activation results despite homology to adjacent target RNA sequences. We suspected that some target RNA sequences might be inaccessible for Cas13a activation due to inherent secondary structure or sequence bias, but it remained unclear how to predict such difficult intrinsic nucleic acid criteria when designing crRNAs. As such, we sought to supplement the demonstrated workflow and shed light on the mechanisms crRNA efficacy.

Here, we tested hundreds of candidate crRNAs for activity bias with Cas13a. Sets of hundreds of crRNAs were tested targeting discrete sequence ranges of the *Y. pestis lcrV* gene that represented regions previously observed to have variable Cas13a detection efficacies. Our intention was to survey sequence regions on a nucleotide by nucleotide basis that corresponded to consistent and inconsistent Cas13a detection to assess why some crRNAs fail to detect a target RNA when others do not. Considering the success of the streamlined crRNA production and test protocols without the loss of SHERLOCK detection sensitivity^23^, we decided to simply assay Cas13a activation to represent the end of the SHERLOCK assay using a high-throughput format via acoustic liquid handler. The results of this effort include thousands of Cas13a activation assays on multiple *lcrV*-derived targets in a number of experimental formats that allowed us to identify characteristics that aid or deter from successful design or implementation of nucleic acid detection strategies. As a result we noted the initial success of the majority of our crRNA (> 91%) regardless of target region or nucleic acid physical properties. We furthermore found that this success could be improved to 97% through altering RNA preparations, target RNA length, and most importantly by changing the commercial source of DNA oligomers used for crRNA preparation. These findings build on previous efforts in the field to underscore the accessibility of CRISPR-Cas protocols for modern biotech applications, and demonstrate the potential scalability of multiplex assays through the use of liquid handling technology and optimized batch reagent preparation protocols implemented without compromising the specificity and sensitivity for which CRISPR-Cas is known.

## Materials and methods

### Design of crRNA

**Figure 1:**
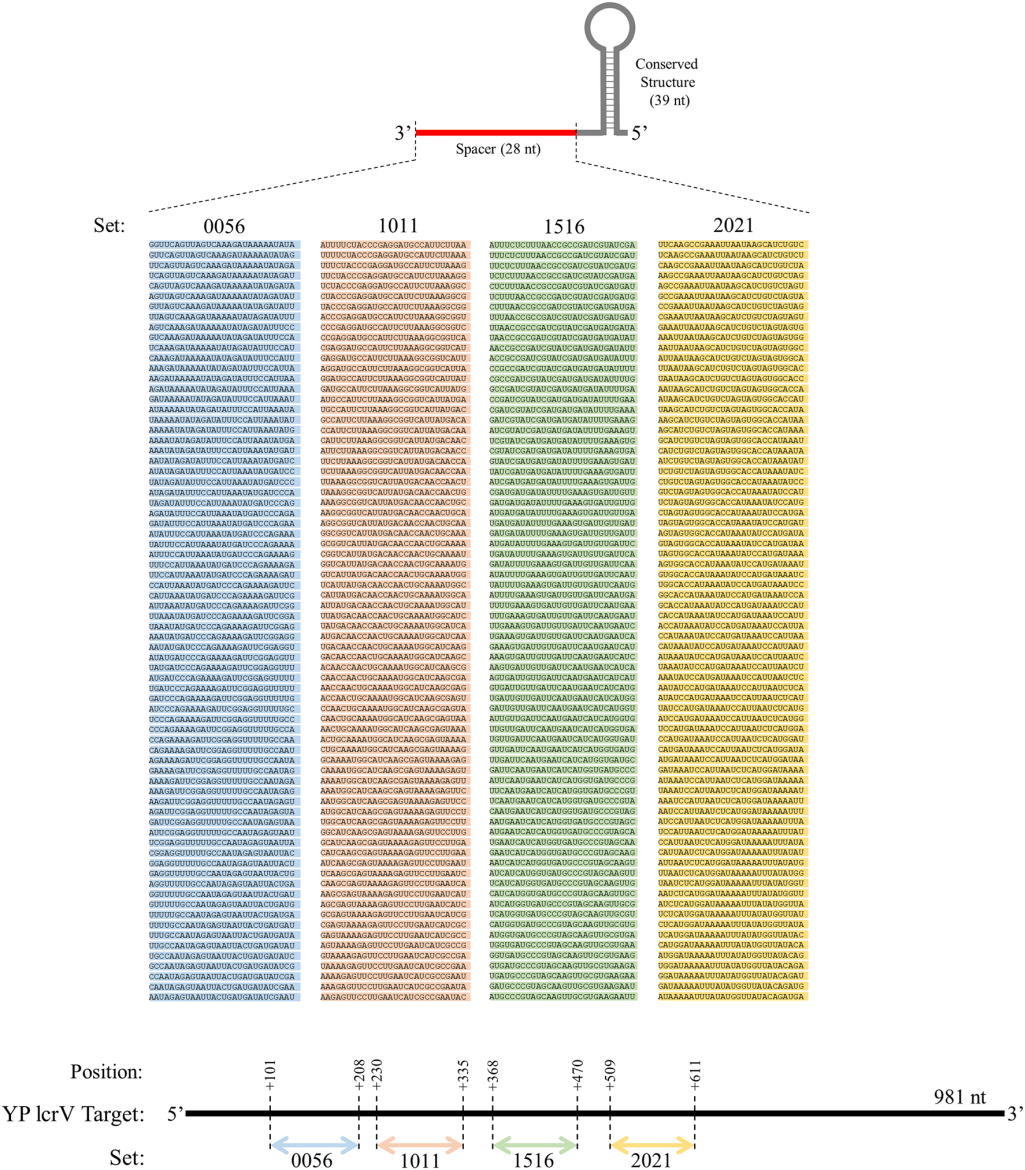
Schematic and sequences of spacer regions for crRNA guide design targeting areas of the *lcrV* mRNA.

The crRNA design scheme was based off of a previously published method targeting the *lcrV* gene from *Y. pestis* strain CO92^23^, where the investigators showed activity from 4 pairs of crRNA with either consistent (cr5/cr6 and cr10/cr11) or inconsistent (cr15/cr16 and cr20/cr21) Cas13a activation. Similarly, each crRNA maintained an architecture consisting of a 39 nt direct repeat for *Leptotrichia wadei* Cas13a (LwaCas13a)^24^ and a 28 nt spacer region targeting the *lcrV* gene. For our purposes, we expanded the regions targeted by the four pairs of previously characterized crRNAs into four regions ranging 102–108 nt in length, within which the spacer sequences of 74 crRNAs were tiled per nucleotide (Fig. 1). Our resulting crRNA set 0056 therefore targeted an expanded region based off of the previously published cr5/cr6, and the same rationale was followed for our crRNA sets 1011 (based off of cr10/cr11), 1516 (based off of cr15/cr16), and 2021 (based off of cr20/cr21).

### Synthesis of crRNA and RNA target sequences

The crRNA molecules were obtained by conducting in vitro transcription (IVT) of DNA oligonucleotides as previously described^23^. Oligonucleotides specific for crRNA contained three parts: the 22 nt T7 promoter, the 39 nt direct repeat for LwaCas13a, and a 28 nt spacer sequence. Each crRNA specific oligonucleotide was combined with another bearing the promoter for the T7 RNA polymerase. The oligonucleotides were purchased from Eurofins Genomics (Louisville, KY) or Integrated DNA Technologies (Coralville, IA) and listed in Supplemental Table 1. IVT was done using the HiScribe T7 Quick High Yield RNA Synthesis Kit (New England Biolabs, Ipswich, MA). The transcription reactions were conducted in high-throughput format using the strategy described in the next section. The individual transcription reactions were performed in 25 μL of total volume. This included 0.5 μL of 100 μM T7 oligonucleotide, 1.5 μL of 100 μM crRNA-encoding DNA oligonucleotide, 1.25 μL of T7 RNA polymerase, 9.25 μL of 2 × NTP buffer and 12.5 μL of nuclease-free ddH2O. The reactions were carried out for 2 h at 37 °C. The resulting crRNAs were used in Cas13a activity assays without additional purification following previously reported protocols for the streamlined production of crRNA while maintaining Cas-associated detection sensitivity^23^.

Target RNA sequences were prepared also using the same HiScribe in vitro transcription system as used for crRNA synthesis. Fragments of the *lcrV* gene from *Yersinia pestis* strain CO92^25^ were amplified using primers listed in Supplemental Table 2 using FastStart Taq DNA polymerase kit (MilliporeSigma, Burlington, MA, USA) according to the manufacturer’s instructions. The forward PCR primers used to amplify *lcrV* fragments included T7 promotor sequences for its incorporation into the amplicons. Two separate fragments of *lcrV* were amplified: the full length version (981 nt) using the primers T7lcrVF and YpestislcrVR and the short version (237 nt, nucleotides 37 to 273 of the gene) containing the target sequences for the first group of crRNAs using the primers RPA56F and RPA56R. Transcription reactions were set up using 2 μL of unpurified DNA amplicon preparation, 2 μL of T7 RNA polymerase, 10 μL of 2 × NTP buffer and 16 μL of nuclease-free ddH2O (30 μL of total reaction volume) and incubated at 37 °C for 2 h, after which 5 μL Turbo DNAse (ThermoFisher, Grand Island, NY) and 15 μL of nuclease-free ddH2O were added (increasing the total volume to 40 μL) and incubated further 30 min at 37 °C to remove the template DNA. The transcript preparations were cleaned up using using RNA Clean and Concentrator 25 kit (Zymo Research, Irvine, CA USA) according to manufacturer’s instructions. RNA concentration was determined using a Qubit fluorometer and RNA BR (broad range) Assay kit (ThermoFisher, Grand Island, NY) and diluted to 50 ng/μL (full length target) or 12 ng/μL (short target) for use in Cas13a activity assays.

### High throughput crRNA performance testing using Echo acoustic liquid handler

Both crRNA synthesis and Cas13a activity assays were conducted using a high throughput workflow in 384 well plates with fluid transfer handled by an Echo 525 acoustic liquid handler (Beckman Coulter, Indianapolis, IN) using the Plate Reformat software provided by the manufacturer.

In order to generate crRNAs in this manner, 64 crRNA transcription reactions were set up using reagent volumes as described above for an individual reaction. This included 62 test crRNAs, a positive control crRNA (set 0056 crRNA 7) and a negative control (crRNA template oligo replaced with TE buffer) as shown in Fig. 1. First, master mix containing all reaction components except for template oligonucleotide was distributed using the Echo from a 6 well Echo qualified Reservoir plate (cat# ER-0050) to an Echo qualified 384 well microplate (cat# ER-0050). 23.5 μL of the master mix was transferred to each well. Subsequently 1.5 μL of the crRNA template oligonucleotides were added to each well containing mastermix using Echo instrument from a previously prepared Echo qualified 384 well microplate. The plates were spun briefly in a centrifuge at approximately 1,500 × *g* to bring all the liquid to the bottom of the wells and remove air bubbles. The plate was sealed using MicroAmp Clear Adhesive Film sealer (ThermoFisher, Grand Island, NY) and incubated for 2 h at 37 °C. After incubation, the plates with transcribed crRNAs were stored in − 80 °C. For long term storage MicroAmp sealers were replaced with Adhesive PCR Sealing Foil (cat# AB-0626, ThermoFisher, Grand Island, NY).

To determine the efficacy of each crRNA, Cas13a nuclease activity assays were conducted using Cas13a enzyme from *L. wadei*^11^ which was synthesized and purified by GenScript Biotech (Piscataway, NJ). The enzyme was stored and diluted in buffer (50 mM Tris–HCl, 600 mM NaCl, 5% Glycerol, 2 mM DTT at pH 7.5). Each nuclease activity assay was performed in 20 μL reaction that included 1 μL of 1 μM Cas13a, 1 μL of 2 μM RNase alert v.2 (from RNaseAlert QC System v2, ThermoFisher, Grand Island, NY), 17.2 μL of nuclease assay buffer (40 mM Tris–HCl, 60 mM NaCl, 6 mM MgCl2, pH 7.3), 0.4 μL of crRNA (from unpurified transcription reaction) and 0.4 μL of target RNA (50 ng/μL for full length target or 12 ng/μL for short target). For each crRNA a total of six reactions were set up, with three target negative reactions and three target positive. First, master mix containing all reaction components except for crRNA and target RNA were distributed using the Echo from 6 well Echo qualified Reservoir plate to a 384 well assay plate (black with clear flat bottom, cat#3762, Corning Life Sciences, Tewksbury, MA). A total volume of 19.2 μL of the master mix was transferred to each well. Next, 0.4 μL crRNAs from previously prepared 384 well microplate were transferred using the Echo to the wells containing the master mix in such a way that each crRNA was added to 6 subsequent wells in the reaction plate (e.g. crRNA from well A1 was added to wells A1-A6 of the reaction plate, crRNA from well A2 was added to wells A7-A12). Finally, 0.4 μL of the target RNAs (previously placed in the area of the crRNA plate not occupied by transcribed crRNAs) were added to three of the wells for each crRNA (e.g. wells A3-A5, A10-A12, and so on). The Cas13a reaction plates were spun briefly in a centrifuge at approximately 1500 × *g* to bring the liquid to the bottom of the wells and remove air bubbles. Immediately after spinning, the reaction plates were sealed using the MicroAmp sealers. The plates were incubated in a Biotek Synergy Neo2 plate reader (Biotek, Winooski, VT) at 37 °C (unless otherwise noted) and fluorescence was read from the bottom of the wells every 5 min for 2 h using excitation at 490 nm, emission at 520 nm and gain set at 100.

All Cas13a assays took place following this protocol using either the full length *lcrV* target RNA of 981 nt or the 237 nt truncated version as noted in each experiment. Experiments involving variations in temperature were assembled by hand to the same reaction concentrations as above, but were incubated in the Bio-Tek plate reader for fluorescence monitoring at the specified reaction temperatures of 37 °C, 42 °C, 47 °C, 52 °C or 57 °C. Other experiments involving temperature variations included the combination and pre-incubation of full-length *lcrV* target RNA and crRNA 0056–07 at 60 °C for 15 min before the addition of Cas13a and buffer components, followed by fluorescence monitoring of the reaction carried out at 37 °C.

### Data analysis

All reactions, control or experimental, were carried out in triplicate. The performance of the crRNA was calculated by subtracting the sum of averages of fluorescence measured for template negative samples over the course of the experiment (25 measurements) from sum of averages for template positive samples. Each sum was normalized to that of an internal control (crRNA 7, set 0056) that was run on each plate. Data was visualized using R package ggplot2^26^. Analysis of variance was carried out where appropriate using rstatix^27^, and principal component analyses were computed with factoextra^28^.

## Results and discussion

### High-throughput screens

The use of Cas13a to recognize difficult pathogens provides an accessible method with high potential for easy adaptation for emerging targets. Successful adaptation, however, depends on the identification of viable crRNAs. While crRNA design criteria exist for various Cas protein activities^17^, we sought to obtain a deeper understanding of the requisite criteria for Cas13a activation. We therefore generated several sets of crRNAs and screened their resultant Cas13a activation potential to this end. Given the recently highlighted shortcomings of peptide-based identification methods against the *lcrV* gene from *Y. pestis*^23^, we used this gene as a template for the design of 296 crRNAs focusing on four distinct regions (Fig. 1). We produced 74 crRNAs from each sub-target area (labeled as crRNA sets 0056, 1011, 1516, 2021) that consisted of 1 nt sliding windows of the 28 nt crRNA spacer sequence. Initial runs were all performed using crRNAs prepared from DNA oligonucleotides obtained from Eurofins Genomics. These sub-target areas represented expanded sequence regions within which previous studies had shown either consistent (represented by sets 0056 and 1011) or inconsistent (represented by sets 1516 and 2021) Cas13a activation via variations in crRNA targeting efficiency^23^. Considering that the SHERLOCK method requires recombinase polymerase amplification (RPA)^29^ that itself is a source of technical variability, we elected to fix target RNA concentrations throughout experiments without RPA to focus on the Cas13a activation potential of each crRNA. This simplification allowed us to observe activity against the 981 nt target RNA in a high throughput format using the LabCyte Echo acoustic liquid handler.

**Figure 2:**
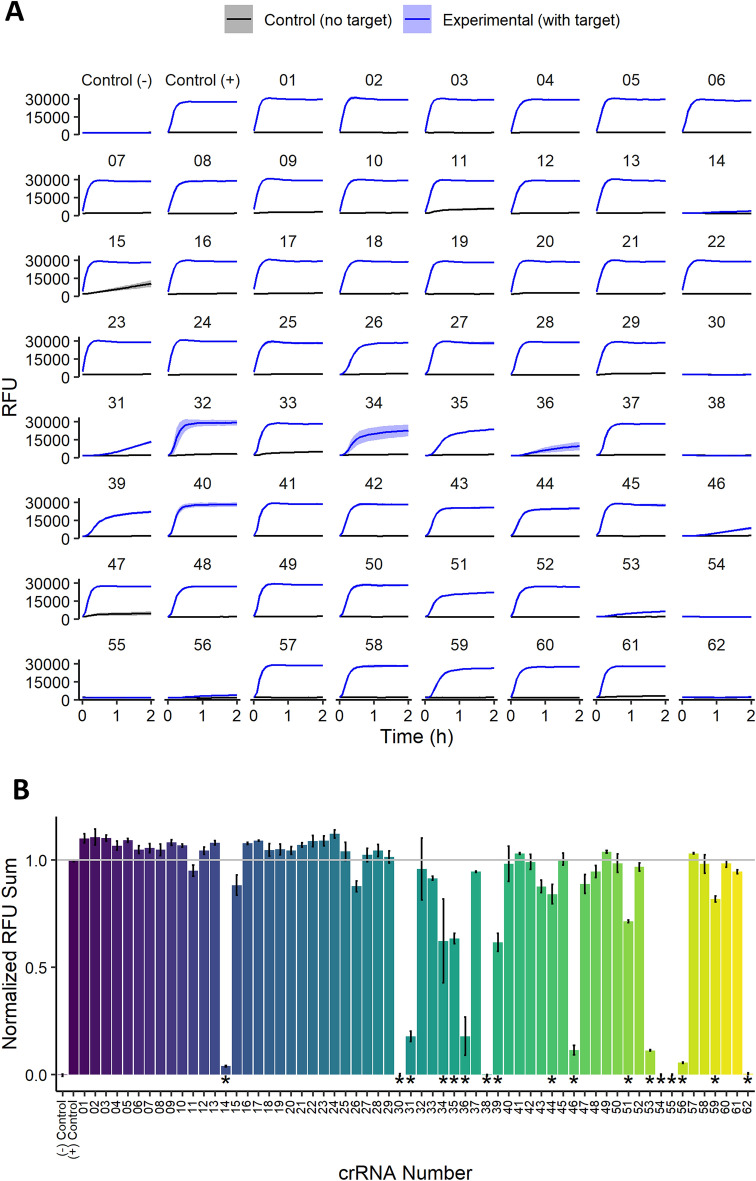
(**A**) Fluorescence traces of Cas13a activation assay for each crRNA from the 0056 set on a single multi-well plate. Solid lines represent mean and shaded regions standard deviation (n = 3). Each crRNA was tested with a control run lacking target RNA refered to as “Control (no target)” and an experimental run refered to as “Experimental (with target)”. Each plate contains an internal negative control lacking crRNA labeled “Control ( −)” and an internal positive control with crRNA 0056–07 labeled “Control ( +)”. (**B**) Summary plot of flourescence throughout the experiment, where the difference in sums per sample between “Control (no target)” and “Experimental (with target)” are normalized against the difference of the internal controls. Bars represent mean values and standard deviation is represented by error bars (n = 3). *p*-values < 0.05 determined by analysis of variance and Tukey test against internal control are denoted by *.

The raw data from each Cas13a activation reaction appeared as fluorescence intensity traces over the 2 h reaction time at 37 °C (Fig. 2A). Two aspects of our high throughput method became immediately apparent upon initial analysis: (1) the assayed crRNAs were stratified into successful (the majority) or unsuccessful groups containing little internal variation, and (2) the maximum fluorescence intensity was reached within 15 min in most successful assays. Our crRNAs only contained 2 nt differences localized to the spacer region, therefore we expected a consistent response from the majority of crRNA targeting a given region. We also expected a similar rate of fluorescence production considering the standardized reaction components (see Materials and Methods).

**Figure 3:**
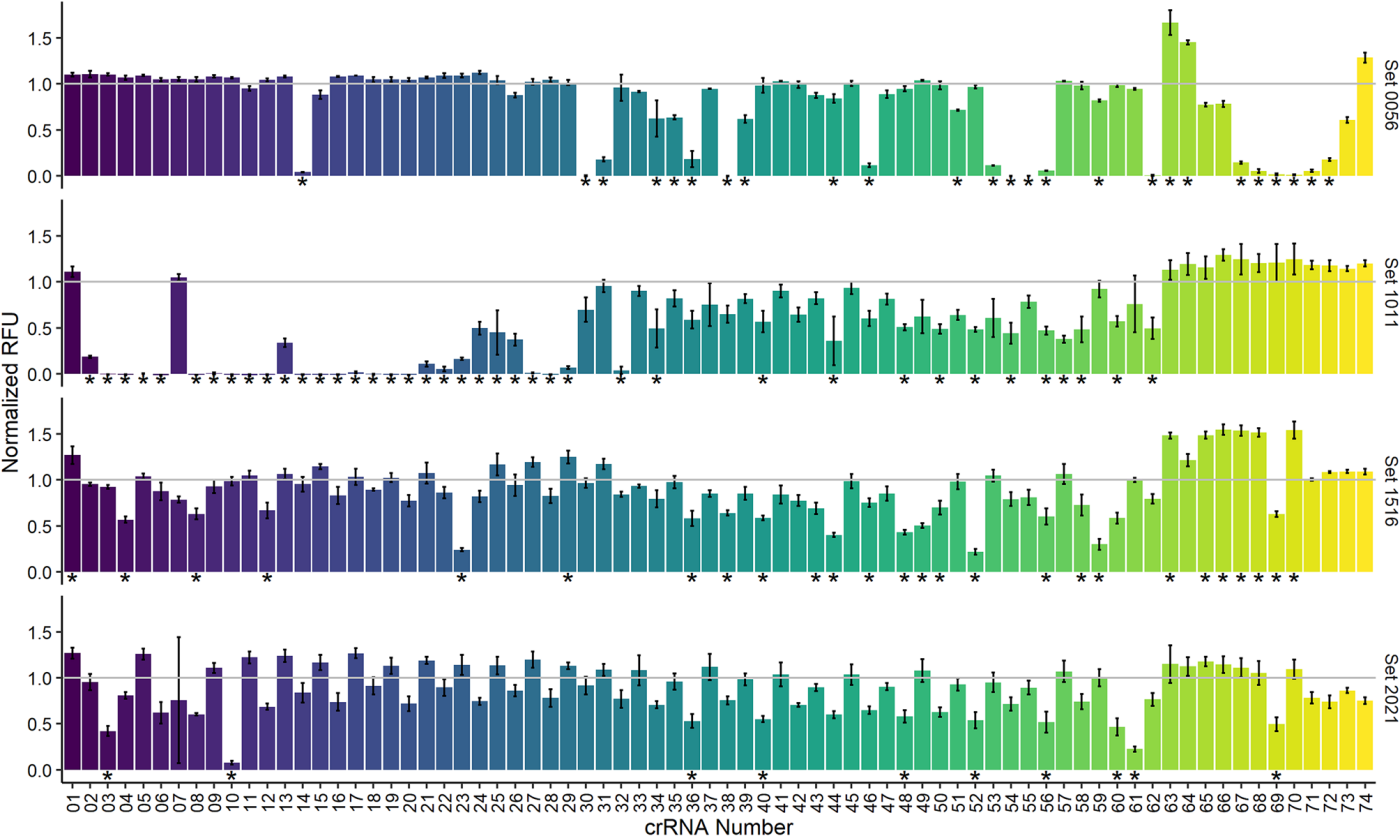
Comparison of Cas13a activation from all tested crRNA across 4 target regions shown as normalized fluorescence sums against internal controls for each set. The crRNA numbers (x-axis) do not correspond to equivalent sequences, but instead crRNA position in their respective series. Bars represent mean and standard deviation is represented by error bars (n = 3), where *p*-values < 0.05 determined by analysis of variance and Tukey test against internal control are denoted by *.

Our assessment of the first set of crRNAs (set 0056), however, also produced unexpected results such as small numbers of inactive crRNAs within groups of active crRNAs. These results were more easily visualized when transforming the data into summary plots and normalizing them to our internal controls (Fig. 2B). Certain samples (crRNAs 14, 30, 31, 36 38, 46, 53, 54, 55, 56 and 62) showed little to no indication of Cas13a activation implying failed target RNA detection. A closer look at the fluorescence traces in Fig. 2A revealed that some samples displayed a slow activation of Cas13a (to be considered “poorly active”: crRNAs 14, 31, 36, 46, 53 and 56) while the others showed no evidence of activation at all (considered “inactive”: crRNAs 30, 38, 54, 55 and 62). We initially attributed this observation to the specific area targeted by crRNA set 0056, however our continued evaluation of the 222 crRNAs in the other 3 target areas produced similar trends (Fig. 3). One particularly unexpected result appeared within crRNA set 1011 where a range of 30 inactive crRNAs contained two with inexplicably high activity. We expected during our experimental design that some crRNAs might target inaccessible sequences representing targeting dead zones, but these observations of differential Cas13a activation did not appear to follow any predictable pattern expected from groups of crRNAs with similar spacer sequences.

When analyzing the fluorescence intensity and the production times of the active, inactive and poorly active subsets, it became apparent that the poorly active subset presented a problem. Cas13a activation should be easily observed considering the standardized reaction components and the high rate of Cas13a hydrolysis, and the resulting quick production of peak fluorescence intensity was observed for most of the effective crRNAs. In fact, these assays were designed such that the only variable affecting fluorescence accumulation would be the concentration of activated Cas13a. This activity is not guaranteed, as previous studies have shown Cas enzymes to be poorly activated due to factors such as misfolded pre-crRNA produced in CRISPR arrays, complementarity of the spacer and target sequences below threshold levels^18^, and target homopolymer preferences^30^. However, our experiments did not involve CRISPR arrays that may have resulted in crRNA misfolding. Additionally, there was a poor likelihood of inactive crRNA secondary structures occurring between adjacently tiled crRNAs which only had a 3% change to nucleotide composition, especially considering that 60% of each crRNA sequence was conserved in all designs (as predicted by T-coffee). We therefore concluded that previous reports of crRNA structure negatively affecting Cas activation were unlikely to explain our observations of adjacent crRNAs in a series with different Cas13a activation capabilities.

We hypothesized that the poor Cas13a activation we observed could arise from a number of sources. One was the RNase contamination of the crRNA that would result in significantly less Cas13a-activation capabilities. Another possible source could have been due to design flaws reducing the complementarity between the crRNAs and target mRNA. A third source of poor Cas13a activation could be the degree of structural heterogeneity of the 981 nt target RNA, wherein multiple complex secondary structures exist in equilibrium that affect the concentration of target RNA with accessible sequences for nucleoprotein complex formation. We therefore designed experiments to further investigate each of these potential sources for inconsistent Cas13a activation.

### crRNA quality

Our first hypothesis to explain poor Cas13a activation was that the quality of specific crRNAs was poor due to low concentrations arising from nuclease contamination. Indeed, RNase contamination is a ubiquitous danger to all RNA experiments. Fortunately, the indicator compound used for our assays was developed as a detection agent for RNase contamination, and it was therefore easy to observe whether our crRNA was contaminated by checking the fluorescence traces of negative control reactions lacking target RNA (Fig. 2A, black traces). Some reactions (crRNAs 11, 29, 33 and 47) showed initial fluorescence increases that did not increase over time, while one (crRNA 15) showed a steady increase throughout the experiment. None of the associated crRNAs, however, fell within the “poorly active” group described above (crRNAs 14, 31, 36, 46, 53 and 56), and all of them showed fluorescence increases when target RNA was present (Fig. 2A, blue traces) that were comparable to the reactions where Cas13a was efficiently active. Therefore, neither the “poorly active” nor “inactive” groups of crRNAs showed evidence of RNA hydrolysis in reactions lacking target RNA, and we concluded that variations in Cas13a activation were not due to poor crRNA quality with respect to RNase contamination.

**Figure 4:**
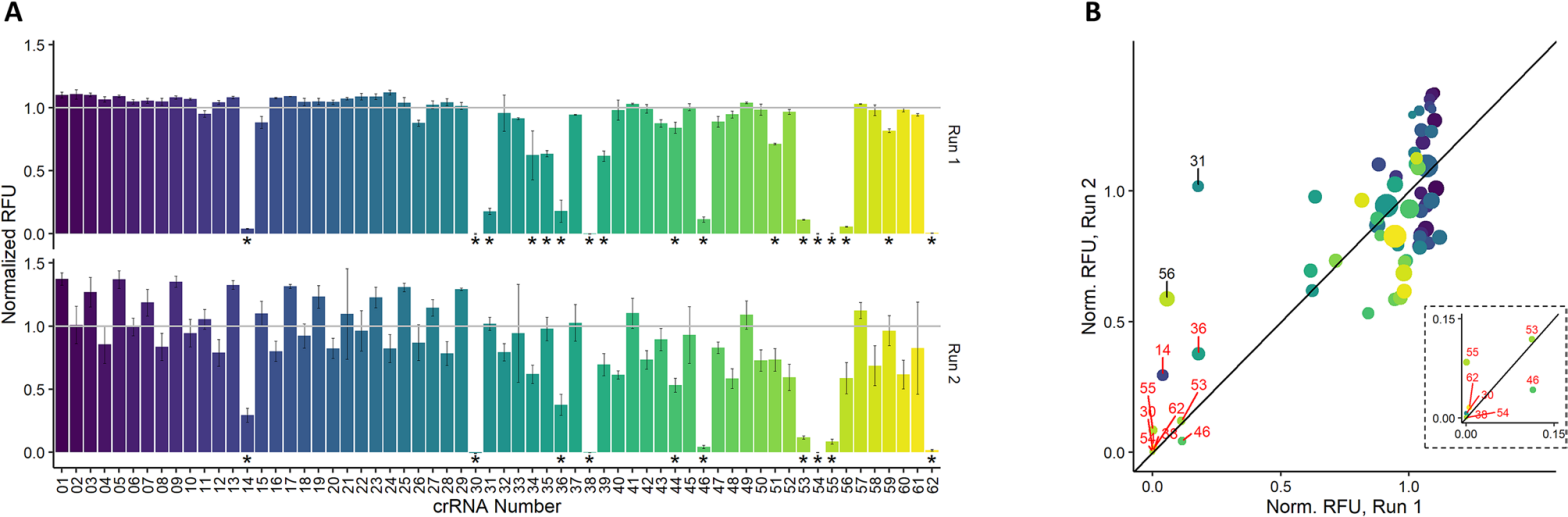
(**A**) Comparison of Cas13a activity from crRNAs from set 0056 prepared in batch on different days, shown as sums of fluorescence values normalized to internal control. Bars represent mean and error bars show standard deviation (n = 3), and *p*-values < 0.05 as determined by analysis of variance and Tukey test against the internal control are denoted by *. (**B**) Comparison of normalized sums of RFU from each crRNA preparation, where values represent mean, dot size represents relative standard deviation, red labels represent crRNAs that produce Cas13a activity significantly different from internal control with *p*-value < 0.05 in both runs, and black labels represent crRNAs where Cas13a activation improved to within 50% of the internal control after repeated preparation.

We noted that poor crRNA quality could also originate from the IVT reactions. While IVT on was carried out in batch on whole crRNA sets, we decided to further pursue this as a possible source of observed variation. When we repeated the synthesis of the 0056 set of crRNAs and ran repeat screens to compare, however, the activity differences between sets was observed to be minimal save few exceptions (Fig. 4). We expected that if the original set of “poorly active” crRNAs suffered from inefficient transcription, we would see these “poorly active” crRNA perform similarly to their neighbors when re-synthesized. However, only 2 of the 11 crRNAs appeared to show this expected increase in activity (Fig. 4B, black labels). While the repeated crRNA preparations did provide more consistent Cas13a activation with regards to the whole 0056 set, this improvement was minimal and recapitulated the inefficiency of the “poorly active” crRNA subset.

### RNA structure and sequence

Nucleic acid structure has been known to convey interesting complexity and activity to specific sequences for a long time^31–33^, but the structures of long sequences are notoriously difficult to predict^34,35^. Considering that nucleic acid secondary structure is thermodynamically governed and that longer sequences tend to contain more possibilities for viable structures, these longer nucleic acids can potentially exist in a diverse structural equilibrium^35^. These RNAs would ideally have a single dominant form characterized by the lowest free energy, however there are many free energy folding algorithms, each with a number of optimizable parameters. As such, with the increasing complexity of long RNAs, these algorithms can predict a number of potential structures with very similar associated free energies, resulting in a number of possible structures. For these reasons, it is difficult to computationally define the exact structure, or even the dominant structure of a long RNA^36^. This structural heterogeneity in the context of a long target RNA (such as our 981 nt target) could easily contribute to varying accessibility of sequence regions, thereby affecting the ability for consistent crRNA binding that ultimately leads to reduced Cas13a activation.

**Figure 5:**
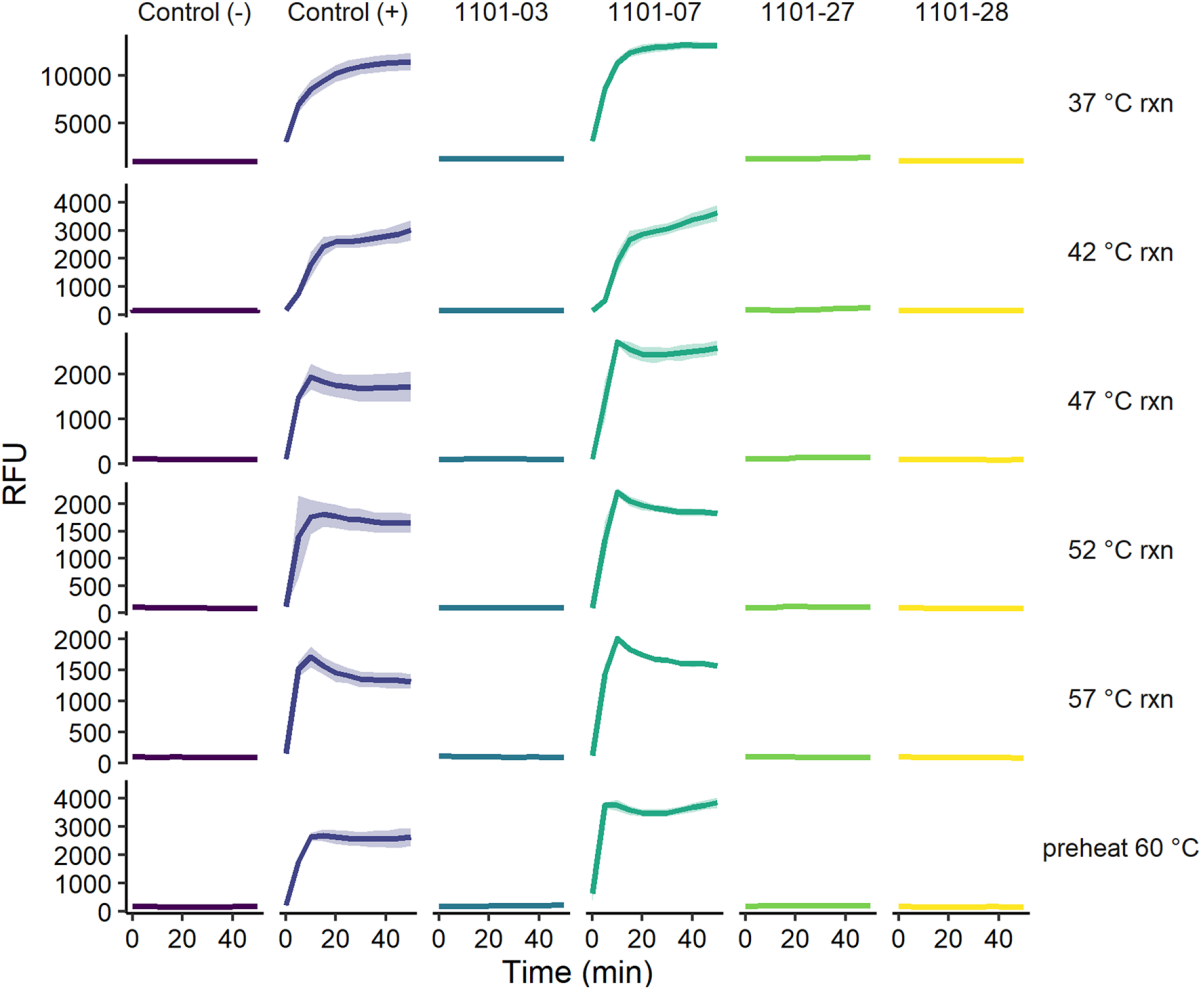
Fluorescence traces of Cas13a experimental variations with increased reaction temperatures (37 °C, 42 °C, 47 °C, 52 °C, 57 °C rxn) and pre-heating of Cas13a + crRNA before addition of target RNA (preheat 60 °C). Solid lines represent mean and shaded regions represent standard deviation (n = 3) of Cas13a activation with selected crRNAs. “Control ( −)” samples contained no crRNA and “Control ( +)” contained the internal standard 0056–07 crRNA.

We decided to alter the structure of our target RNA to test whether there were any structural effects on Cas13a activation. As any denaturant meant for the target RNA would similarly affect the crRNA and protein, we sought the use of heat as a mild and accessible option to incrementally alter nucleic acid secondary structure. Keeping in mind that protein stability could not be compromised, we utilized incremental increases in reaction temperature with the expectation that they might provide structural changes to present previously inaccessible regions of the target RNA. Considering that the crRNAs in question appeared inactive only in narrow sequence windows with respect to the target RNA, we believed any existing secondary structure affecting Cas13a activation would not require extensive denaturation for its disruption. Four crRNAs were selected for these assays that were complementary to sequences within the 1011 region, three of which had been classified “inactive” (crRNAs 1101-03, 1101-27 and 1101-28), and one that was “active” (crRNA 1101-07). Reactions were prepared by hand at room temperature and then incubated for the Cas13a assay at the specified temperatures (Fig. 5). While the maximum fluorescence at increased incubation temperatures was lower than those from 37 °C, we observed no temperature effects on overall Cas13a activation. Interestingly, incubation 20 °C above ideal temperatures appeared to neither denature protein nor crRNA enough to disrupt hydrolysis activity in control reactions. The combination of reagents at room temperature and induced protein conformational change^37^ therefore appeared to provide the nucleoprotein complex with stability against the subsequent temperature changes. Considering our relative abundance of reagents in the reaction (~ 20-fold more Cas13a and ~ 300-fold more crRNA than target RNA), all of the Cas13a should ideally be ready to seek the target RNA, which was set as the limiting reagent for the reaction. Therefore these reaction conditions showed no evidence of changing target RNA binding.

We reasoned that if the incremental increases in reaction temperature were not enough to nudge the potentially inhibitory target RNA structure into an accessible one, then perhaps it may be accomplished by pre-heating RNA before combining with Cas13a. We therefore pre-heated both target RNA and crRNA together at 60 °C for 15 min before the addition of Cas13a and incubation at 37 °C. Again, we observed no changes in Cas13a activation. Similar to previous temperature experiments, the observed fluorescence intensities were lower than reactions carried out at 37 °C, but Cas13a activation was concordant with other reactions at this temperature. Considering that all structural changes and molecular associations took place in a cooling solution (37 °C at the lowest) with no incubation time before analysis, we were intrigued to find that the RNA folding, protein conformational change, and complex association steps were still successful. While previous investigators have shown small temperature changes are enough to significantly alter RNA structural equilibrium between ON and OFF states related to downstream activity^38^, our results suggested that temperature changes would not be able to rescue the inability to activate Cas13a by these crRNAs. Interestingly, our results also showed the resilience of the Cas13a assay to suboptimal reaction conditions, wherein Cas13a activity could still be observed after dramatic changes in both reaction temperature as well as reaction preparation.

### RNA sequence

**Figure 6:**
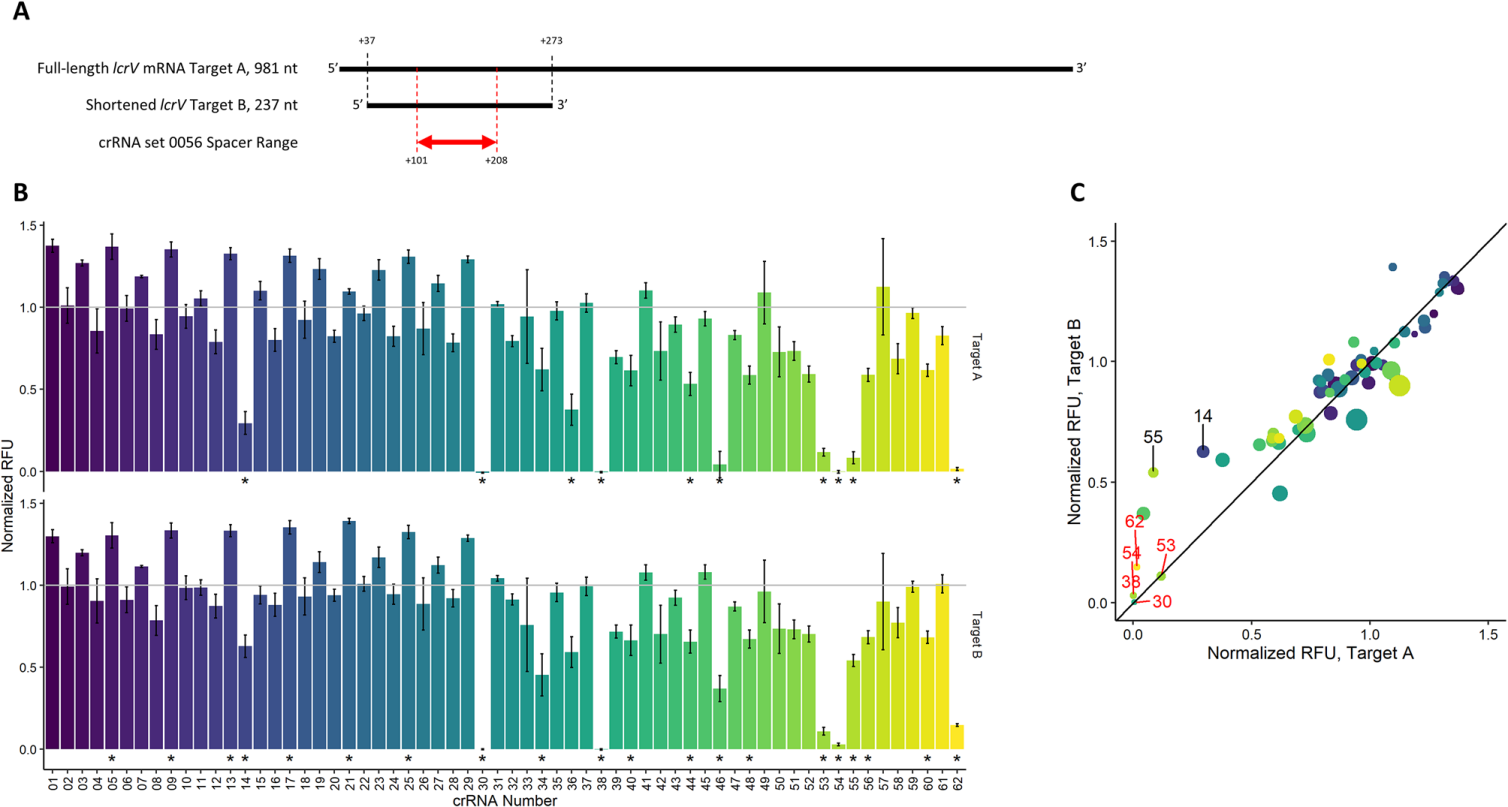
(**A**) Schematic of *lcrV* RNA of different lengths and the complementary region associated with crRNA set 0056. (**B**) Comparison of fluorescence sums normalized to internal control between different target lengths (Target A and Target B, as defined in panel A), where bars represent mean and error bars represent standard deviation (n = 3). (**C**) Comparison of normalized fluorescence sums from different target regions, where values represent mean, dot size represents relative standard deviation (n = 3), red labels represent crRNAs bearing unimproved activity (*p*-value < 0.5 as determined by analysis of variance and Tukey test against internal control) against both target lengths, and black labels represent Cas13a activation from crRNA that improved to within 50% of internal control after shortening target length.

We continued to probe the structural limitations of crRNA targeting by redesigning our target RNA. The target up to this point had been the transcribed 981 nt *Y. pestis* lrcV gene, and previous experiments had indicated that if target RNA structure was altering the Cas13a activation potential of similar crRNAs, then that structure was not easily disrupted with heat. We therefore shortened our target RNA to a 237 nt section within the 0056 region of the *lcrV* gene (Fcigure 6A). As discussed above, this set contained 8 crRNAs that continued to display poor Cas13a activation after repeating assays with freshly prepared RNA. We therefore repeated crRNA preparation and carried out assays against the new shortened target (denoted as “Target B” in Fig. 6), and observed that most of the questionable crRNA continued to perform poorly compared to their neighbors (Fig. 6B,C). On the other hand, we did note crRNAs 0056-14 and 0056-55 induced Cas13a activation similar to their neighbors, bringing the number of “inactive” crRNAs down to 6 for the tested set. Considering the lack of Cas13a activation recovery from repeated RNA preparations, Cas13a assay conditions, and target RNA length, we concluded the Cas13a variations may not have been the result of target RNA structure.

**Figure 7:**
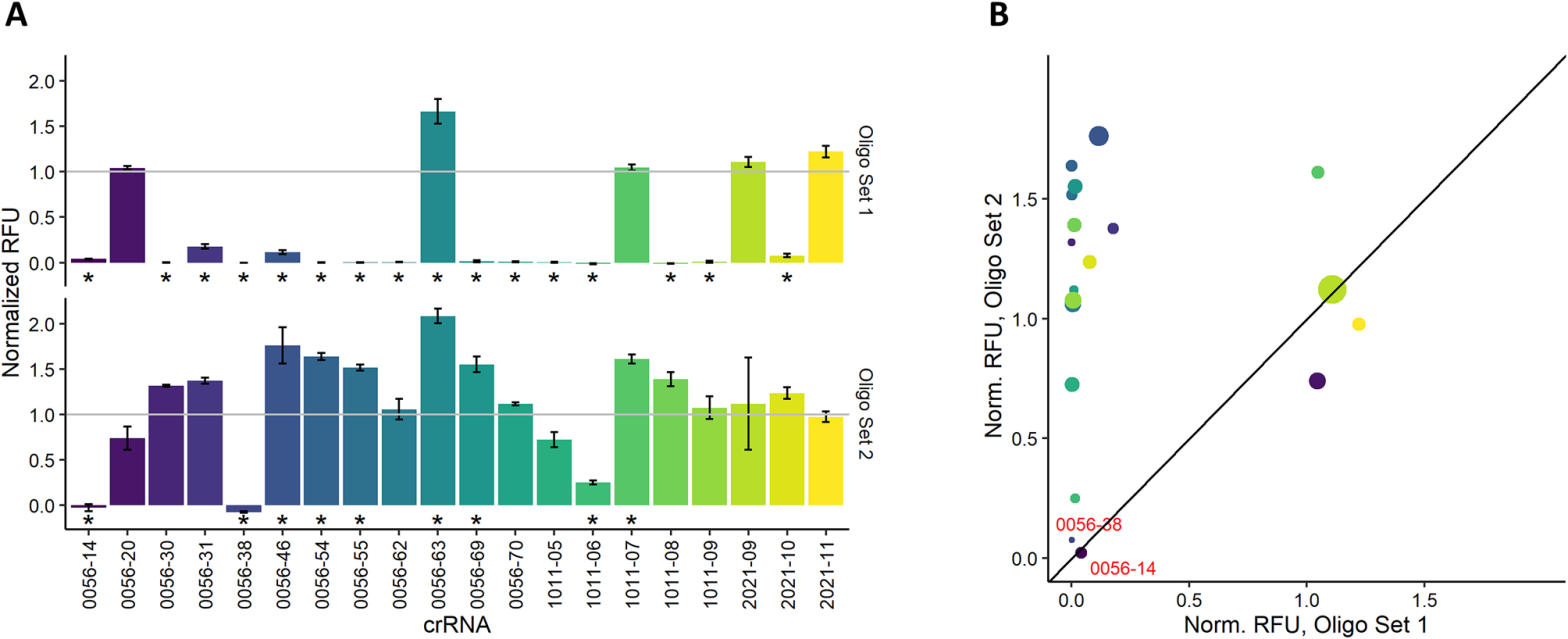
(**A**) Comparison of fluorescence sums normalized to internal control crRNA when crRNA are prepared from DNA oligos from different companies, where bars represent mean and error bars represent standard deviation (n = 3). (**B**) Comparison of normalized fluorescence sums via dot plot where red labels represent crRNAs with *p*-value < 0.05 as determined by analysis of variance and Tukey test against internal controls that remained poorly active despite DNA oligo source.

We then moved to consider the possibility that RNA sequence fidelity was influencing Cas13a activation. Our crRNA sequences were designed based on the isolated *lcrV* gene^23^, which was generated via IVT for all experiments. We confirmed the sequence of this gene after multiple rounds of PCR replication to produce the IVT template, and this confirmation led us to again focus on the quality of our crRNA. While we had shown there was no RNase contamination in crRNA preparations, and that our IVT batch reactions resulted in no issues preparing full crRNA sets, we did observe that different preparations of crRNA displayed increases in Cas13a activation. Considering our protocols, we decided there was a possibility the increase in Cas13a activation between preparations was the result of poor IVT template quality. While all DNA oligomers used as templates for IVT up to this point had been ordered together dispensed in 96 well plates, and therefore had all progressed through the same lot of commercial QC checks, these oligomers were the only unique sample-to-sample variable among our experiments. We therefore chose to order DNA for 20 specific crRNA IVT templates from a different DNA synthesis company and repeat the analysis. Oligos for the synthesis of crRNA 0056-20, 0056-63, 1011-07, 2021-09, and 2021-11 represented 5 “active” reactions to use as positive controls. The other crRNAs (0056-14, 0056-30, 0056-31, 0056-38, 0056-46, 0056-54, 0056-62, 0056-69, 0056-70, 1101-05, 1101-06, 1101-08, 1101-09 and 2021-10) represented 15 crRNAs that previously were either “poorly active” or “inactive”. We used these oligos to perform batch IVT using the same methods as before and observed large differences in Cas13a activity (Fig. 7). Of the 20 tested crRNAs, only 2 remained inactive when comparing oligo templates from both sources (crRNAs 0056-38 and 0056-14). The other 18 crRNAs induced appreciable Cas13a activation, whereas the majority (15 of the 20) were comparable to the internal standard crRNA 0056-07. Previous experiments had shown that alterations in RNA preparation, Cas13a assay conditions, and target RNA length all affected Cas13a activation to some degree, but repeat experiments showed the importance of DNA quality to the success of the Cas13a assay. While the renewed Cas13a activity from our methods did not account for 100% success of our tested crRNAs, we were able to conclude that our largest variable in the design of successful crRNAs was the source (and/or unknown handling steps) relating to commercially procured DNA that initiated our workflow.

Previous studies have developed algorithms to predict the potential of crRNAs to successfully activate Cas13a for emerging target RNA detection capabilities^22^ (and reviewed in^39^). While algorithm development for crRNA performance prediction can indeed be useful, we believe the true value of a design tool to be the target identification capabilities of broad-targeting crRNAs across sequence diversity with minimal experimental input. With this application in mind, the above-mentioned tools in addition to unpublished ones such the ADAPT algorithm^20^ show promise in minimizing the number of crRNAs required to span the phylogenetic diversity of emerging bacterial and viral species. Building from previous structural and activity studies on various Cas proteins, the guidelines for mature crRNA design employed in this study are streamlined. Our experiments furthermore appear to indicate that finding the “perfect” target sequence for crRNA design is relatively unimportant, allowing design algorithms that can focus on capturing phylogenetic diversity. While we tested neither a variety of targets nor an extensive set of crRNAs, we did note that the systematic production of 296 crRNAs varying only within the 28 nt spacer region of a 67 nt shell and targeting 4 distinct regions of a single 981 nt RNA resulted in successful Cas13a activation in > 91% of experiments without optimization (271 of 296 crRNAs, Fig. 3). Of the 271 successful crRNAs, 21 of these (~ 7.7%) showed relatively “poor” Cas13a activation, but activation nonetheless. The fluorescence signals produced by each crRNA set varied slightly with the occurrence of each experiment (Fig. S1A), however normalization to internal standards allowed us to conclude that the target regions used had minimal impact on Cas13a activation potential despite the intrinsic and unpredictable RNA structure of the target RNA (Fig. S1B). The Cas13a activation variations we did observe were subsequently determined to be independent of both experimental variability (Fig. S1C) and crRNA physical properties (Fig. S1D). We showed in addition to the success of the assayed crRNAs that Cas13a activation could still be improved through experimental optimizations, and that success was achievable through a variety of experimental conditions leaving us with 287 viable crRNAs out of 296 (~ 97%). Finally, we highlighted the impact of high quality DNA on the success of any experiment.

## Conclusions

Our high throughput methods allowed us to probe previously reported variable Cas13a activation localized to specific target sequence regions. We found, however, that expanding the scope of targeted sequence regions and tiling large sets of crRNAs per nucleotide against different length versions of the *lcrV* gene resulted in successful Cas13a activation the majority of the time. We were able to further improve the viability of crRNA designs by altering the source of commercial DNA used in RNA preparation, for 70% of the crRNAs derived from one vendor’s oligonucleotides were comparable to internal controls where cognate crRNAs derived from a different vendor’s oligonucleotides failed. This was not a systematic study between different commercial sources, and variations could be due to unknown factors associated with shipment, storage and handling of different DNA oligo batches. We therefore noted a successful Cas13a activation from of 97% our tested crRNA. While it is unclear why the remaining 3% of crRNA did not successfully detect the *lcrV* target, we did note an improvement of target detection when altering target length that was not observed when altering DNA vendor, suggesting that target RNA may remain a factor for the ultimate Cas13a activity.

The feasibility of generating large amounts of reproducible data with minimal user input further improves each day. Here, we have shown that by employing Acoustic Liquid Handling technology we continued to increase workflow efficiency of Cas13a assays building on previous work^23^, which provided us with wide-scoped snapshots of the targetable areas of a chosen RNA. We believe that this workflow still maintains the potential for massive scale-up. Considering the success of our crRNA screens, extending the analysis to identify members of a sequence-diverse group of targets could be as simple as modifying our current workflow to multiplex a variety of crRNA combinations across multiple experiments, so long as the crRNA designs were made by competent evaluation of high fidelity target sequences and the initial input reagents for RNA production maintained high quality standards. While emergent target sequences are not always predictably available, the use of such screening methods could provide the basis for the development of rapid and specific nucleic acid identification protocols. While our data indicated neither sequence bias towards either crRNA design nor target RNA sequence to preclude Cas13a activation, the assembly and assessment of large reaction pools were achievable in a minimal amount of time. This efficiency allowed us the opportunity to probe a number of different variables wherein the longest steps were the 2 h IVT and Cas13a reactions, respectively. The deployment of derivatives of this method in addition to associated scale-up is therefore feasible where the limiting aspect seems to only be the preliminary identification of a target. Given the existence of 64 defined sequence variations of a specific target, one could follow our method to design crRNAs and perform a single sample screen of all variants on a single 384-well plate (in triplicate, including controls) less than half a day. Compared to the production times of antibodies and their efficacy shortfalls, our data reinforce the power of modern nucleic acid identification methods that, coinciding with the numerous examples of associated sequence discrimination capabilities in the last decade, indicate the value of a concerted direction and organization for the continued implementation of wide-scoped diagnostic tools.

## Supplementary Information

Supplementary Information 1.

Supplementary Information 2.

Supplementary Information 3.

## Data Availability

The datasets generated and analyzed during the current study are available in supplemental information, as well as the sequences of all DNA oligos used to generate RNA.

## References

[1] Ishino Y, Krupovic M, Forterre P (2018). History of CRISPR-Cas from encounter with a mysterious repeated sequence to genome editing technology. J. Bacteriol..

[2] Ding W, Zhang Y, Shi S (2020). Development and application of CRISPR/Cas in microbial biotechnology. Front. Bioeng. Biotechnol..

[3] Finger-Bou M, Orsi E, van der Oost J, Staals RH (2020). CRISPR with a happy ending: Non-templated DNA repair for prokaryotic genome engineering. Biotechnol. J..

[4] Rönspies M, Schindele P, Puchta H (2021). CRISPR/Cas-mediated chromosome engineering: Opening up a new avenue for plant breeding. J. Exp. Bot..

[5] Tyagi S, Kumar R, Kumar V, Won SY, Shukla P (2021). Engineering disease resistant plants through CRISPR-Cas9 technology. GM Crops Food.

[6] Xie, S., Ji, Z., Suo, T., Li, B. & Zhang, X. Advancing sensing technology with CRISPR: from the detection of nucleic acids to a broad range of analytes—A Review. *Anal. Chim. Acta*, 338848 (2021). 10.1016/j.aca.2021.338848 34711331

[7] Frangoul H (2021). CRISPR-Cas9 gene editing for sickle cell disease and β-thalassemia. N. Engl. J. Med..

[8] Wang T (2021). Sequential CRISPR gene editing in human iPSCs charts the clonal evolution of myeloid leukemia and identifies early disease targets. Cell Stem Cell.

[9] Broughton JP (2020). CRISPR–Cas12-based detection of SARS-CoV-2. Nat. Biotechnol..

[10] Makarova KS (2020). Evolutionary classification of CRISPR–Cas systems: a burst of class 2 and derived variants. Nat. Rev. Microbiol..

[11] Gootenberg JS (2017). Nucleic acid detection with CRISPR-Cas13a/C2c2. Science.

[12] Park JS (2021). Digital CRISPR/Cas-assisted assay for rapid and sensitive detection of SARS-CoV-2. Adv. Sci..

[13] Chen, F.-E. *et al.* Point-of-Care CRISPR-Cas-assisted SARS-CoV-2 detection in an automated and portable droplet magnetofluidic device. *Biosens. Bioelectron.* 113390 (2021). 10.1016/j.bios.2021.113390 PMC8170879 34171821

[14] de Puig H (2021). Minimally instrumented SHERLOCK (miSHERLOCK) for CRISPR-based point-of-care diagnosis of SARS-CoV-2 and emerging variants. Sci. Adv.

[15] Dara M, Talebzadeh M (2020). CRISPR/Cas as a potential diagnosis technique for COVID-19. Avicenna J. Med. Biotechnol..

[16] Kaminski MM, Abudayyeh OO, Gootenberg JS, Zhang F, Collins JJ (2021). CRISPR-based diagnostics. Nat. Biomed. Eng..

[17] Creutzburg SCA (2020). Good guide, bad guide: spacer sequence-dependent cleavage efficiency of Cas12a. Nucleic Acids Res..

[18] Liu L (2017). The molecular architecture for RNA-guided RNA cleavage by Cas13a. Cell.

[19] Lino CA, Harper JC, Carney JP, Timlin JA (2018). Delivering CRISPR: A review of the challenges and approaches. Drug Deliv..

[20] Metsky, H. C., Freije, C. A., Kosoko-Thoroddsen, T.-S. F., Sabeti, P. C. & Myhrvold, C. CRISPR-based surveillance for COVID-19 using genomically-comprehensive machine learning design. *bioRxiv*, 2020.2002.2026.967026. 10.1101/2020.02.26.967026 (2020).

[21] Ackerman CM (2020). Massively multiplexed nucleic acid detection with Cas13. Nature.

[22] Jiang W (2015). Cas9-assisted targeting of chromosome segments CATCH enables one-step targeted cloning of large gene clusters. Nat. Commun..

[23] Schultzhaus Z, Wang Z, Stenger D (2021). Systematic analysis, identification, and use of CRISPR/Cas13a-associated crRNAs for sensitive and specific detection of the lcrV gene of Yersinia pestis. Diagn. Microbiol. Infect. Dis..

[24] Myhrvold C (2018). Field-deployable viral diagnostics using CRISPR-Cas13. Science.

[25] Mulvaney SP (2018). Rapid design and fielding of four diagnostic technologies in Sierra Leone, Thailand, Peru, and Australia: Successes and challenges faced introducing these biosensors. Sens. Bio-Sens. Res..

[26] Wickham H (2011). ggplot2. Wiley Interdiscip. Rev. Comput. Stat..

[27] Kassambara, A. rstatix: Pipe-friendly framework for basic statistical tests. *R package version 0.6. 0* (2020).

[28] Kassambara, A. & Mundt, F. Package ‘factoextra’. *Extract and visualize the results of multivariate data analyses* **76** (2017).

[29] Kellner MJ, Koob JG, Gootenberg JS, Abudayyeh OO, Zhang F (2019). SHERLOCK: Nucleic acid detection with CRISPR nucleases. Nat. Protoc..

[30] East-Seletsky A, O'Connell MR, Burstein D, Knott GJ, Doudna JA (2017). RNA targeting by functionally orthogonal type VI-A CRISPR-Cas enzymes. Mol. Cell.

[31] Li N, Huang F (2005). Ribozyme-catalyzed aminoacylation from CoA thioesters. Biochemistry.

[32] Doherty EA, Doudna JA (2001). Ribozyme structures and mechanisms. Annu. Rev. Biophys. Biomol. Struct..

[33] Breaker RR (2011). Prospects for riboswitch discovery and analysis. Mol. Cell.

[34] Miao Z, Westhof E (2017). RNA structure: Advances and assessment of 3D structure prediction. Annu. Rev. Biophys..

[35] Bevilacqua PC, Ritchey LE, Su Z, Assmann SM (2016). Genome-wide analysis of RNA secondary structure. Annu. Rev. Genet..

[36] Lee J (2014). RNA design rules from a massive open laboratory. Proc. Natl. Acad. Sci. USA.

[37] Jackson RN, van Erp PB, Sternberg SH, Wiedenheft B (2017). Conformational regulation of CRISPR-associated nucleases. Curr. Opin. Microbiol..

[38] Chowdhury S, Ragaz C, Kreuger E, Narberhaus F (2003). Temperature-controlled structural alterations of an RNA thermometer. J. Biol. Chem..

[39] Cui Y, Xu J, Cheng M, Liao X, Peng S (2018). Review of CRISPR/Cas9 sgRNA design tools. Interdiscip. Sci. Comput. Life Sci..

