# Supplementary material for: Large scale screening of CRISPR guide RNAs using an optimized high throughput robotics system
Source: Sci Rep. 2022 Aug 17;12:13953. doi: 10.1038/s41598-022-17474-8 (PMC9385653; doi:10.1038/s41598-022-17474-8)
Supplement: Supplementary file 3 — Supplementary Information 3. [file 41598_2022_17474_MOESM3_ESM.docx]

**Supplemental Files**


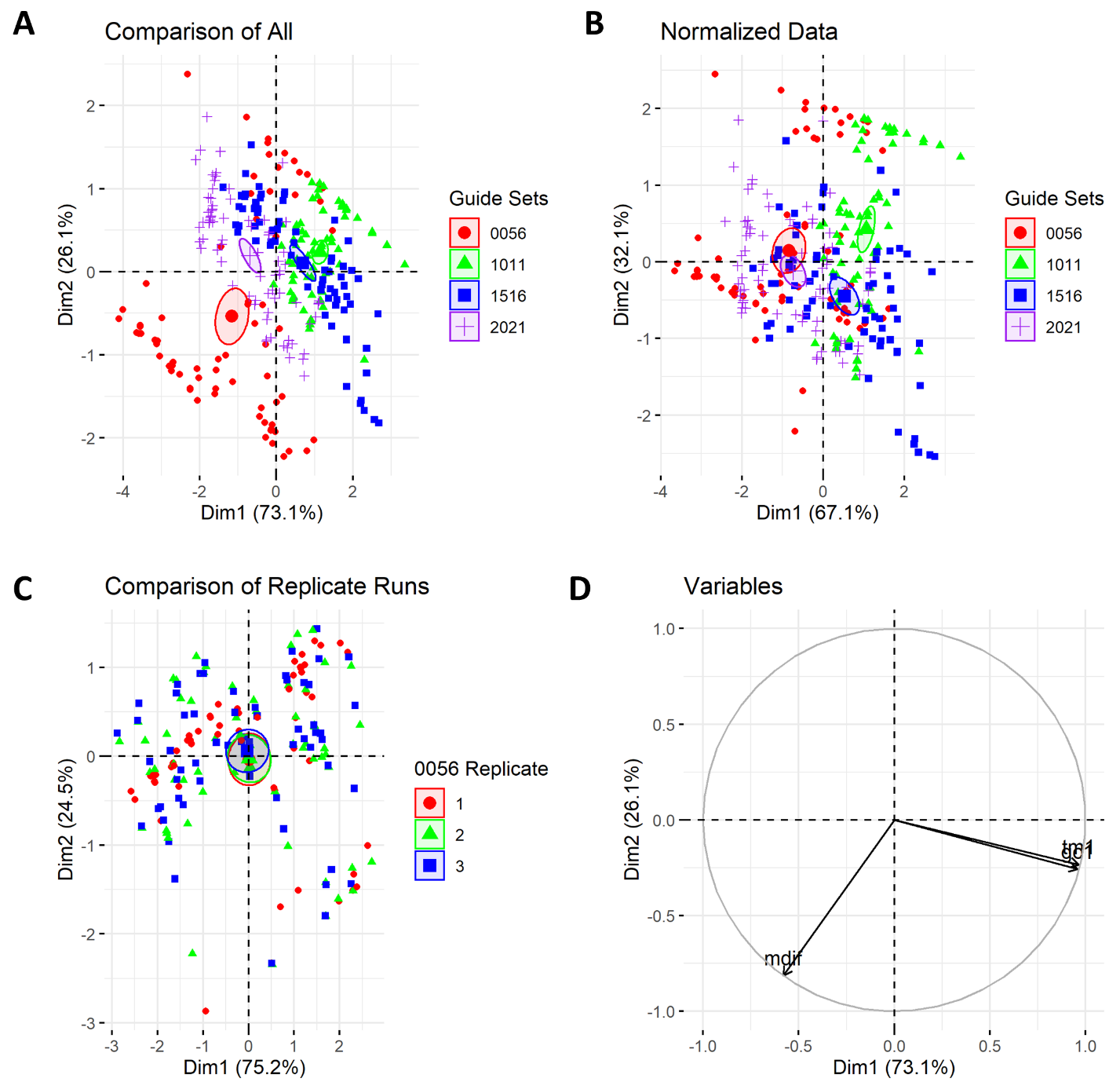


Figure S1. Principal component analyses comparing (A) Cas13a activity of all guide sets, (B) activity of all guide sets normalized to internal controls, (C) Cas13a activity within a single guide set in replicate runs, and (D) Cas13a activity (mdif) with respect to melting temperature (tm1) and GC content (gc) of each guide.

*
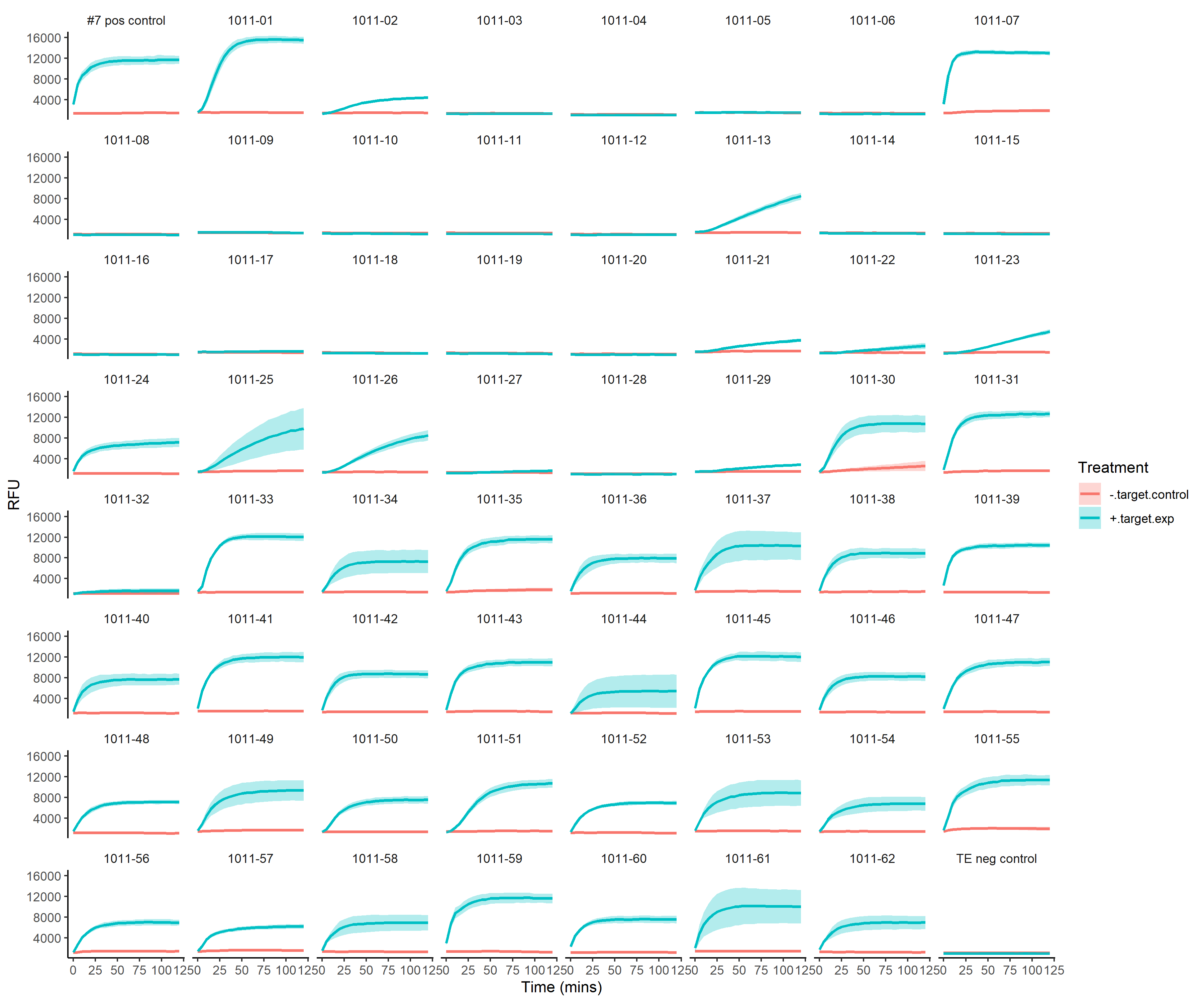
*

Figure S2. Fluorescence traces of guide set 1011 with controls of no target (red) or no guide (TE neg control) over time.

*
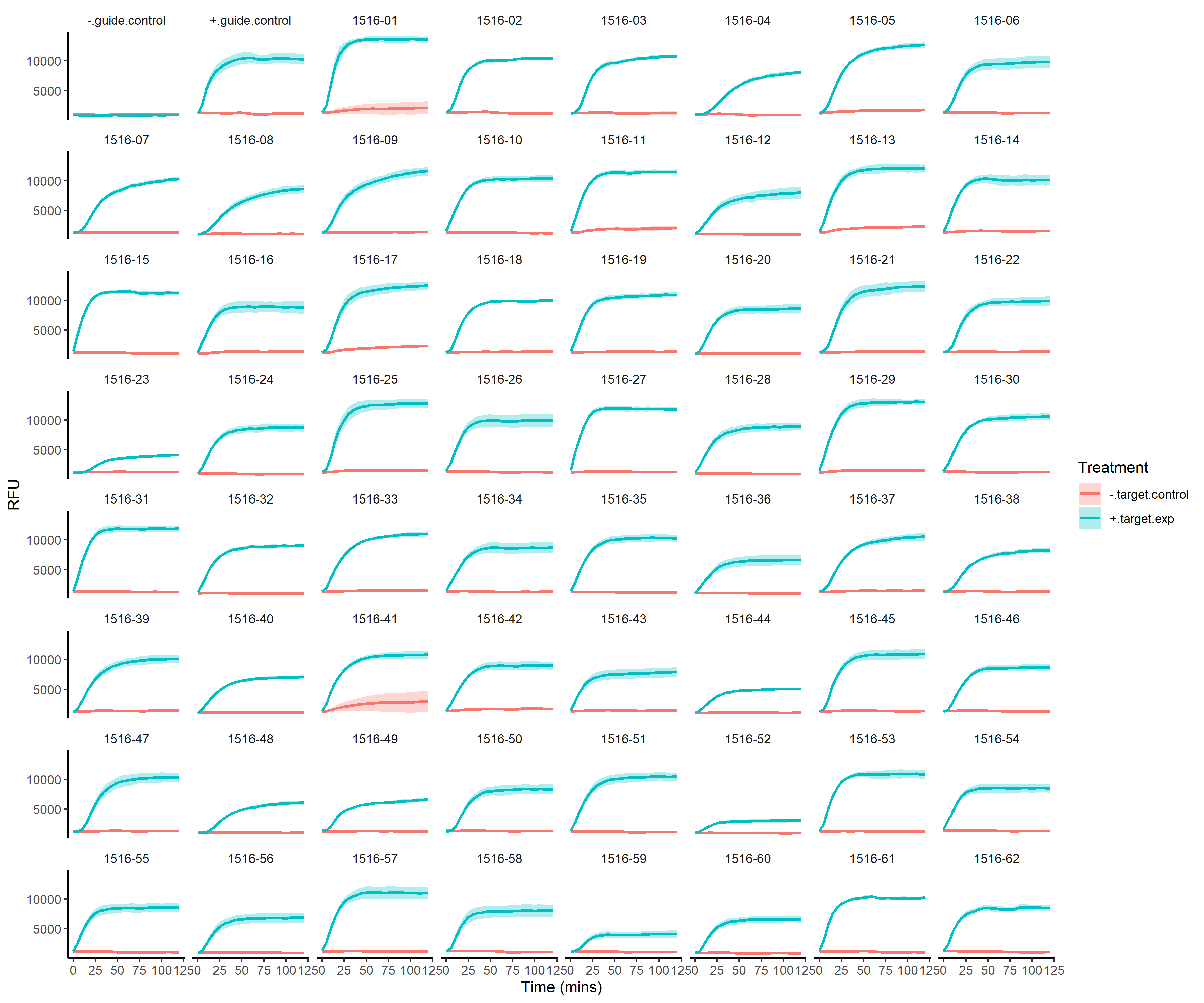
*

Figure S3. Fluorescence traces of guide set 1516 with controls of no target (red) or no guide (TE neg control) over time.


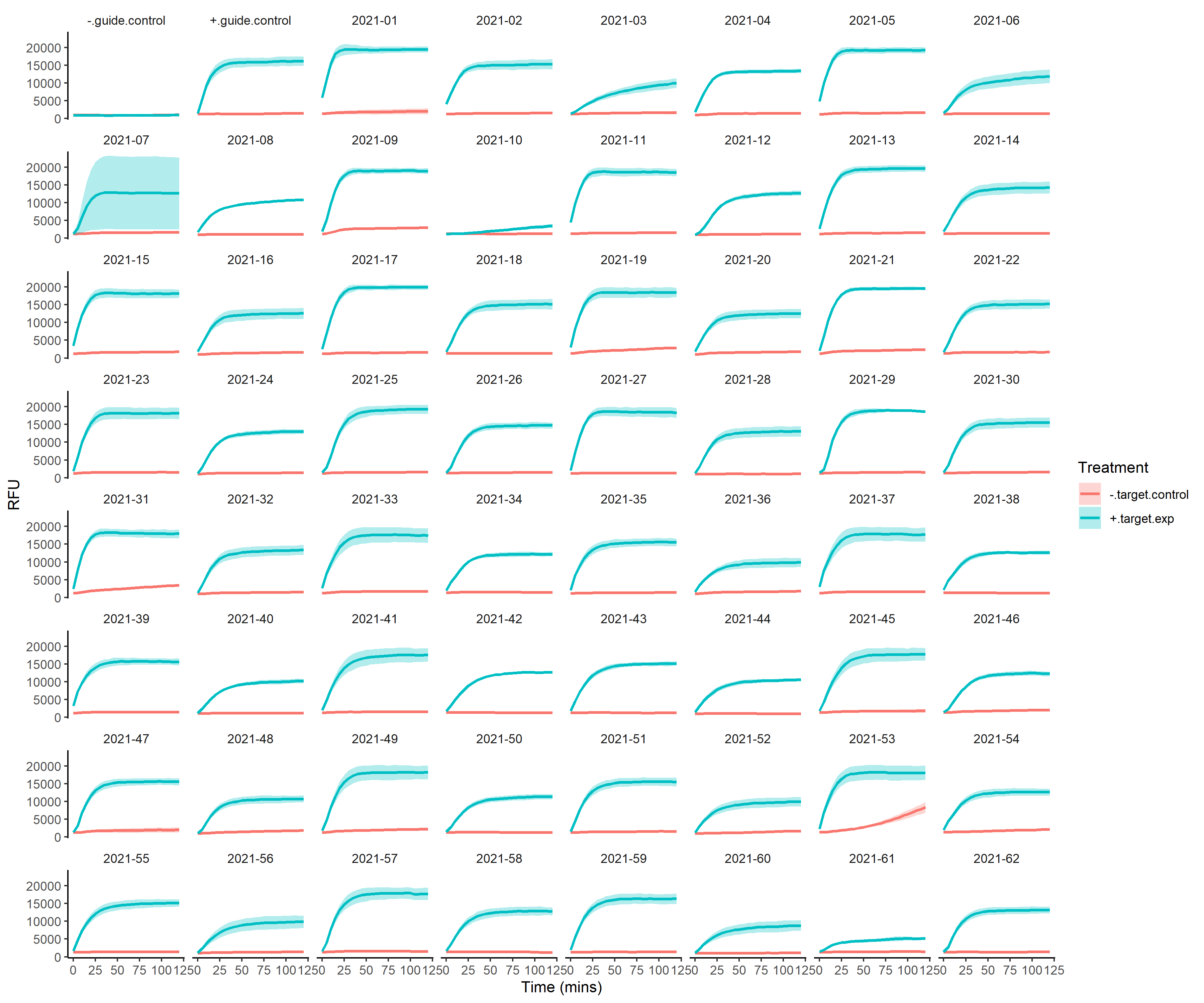


Figure S4. Fluorescence traces of guide set 2021 with controls of no target (red) or no guide (TE neg control) over time.


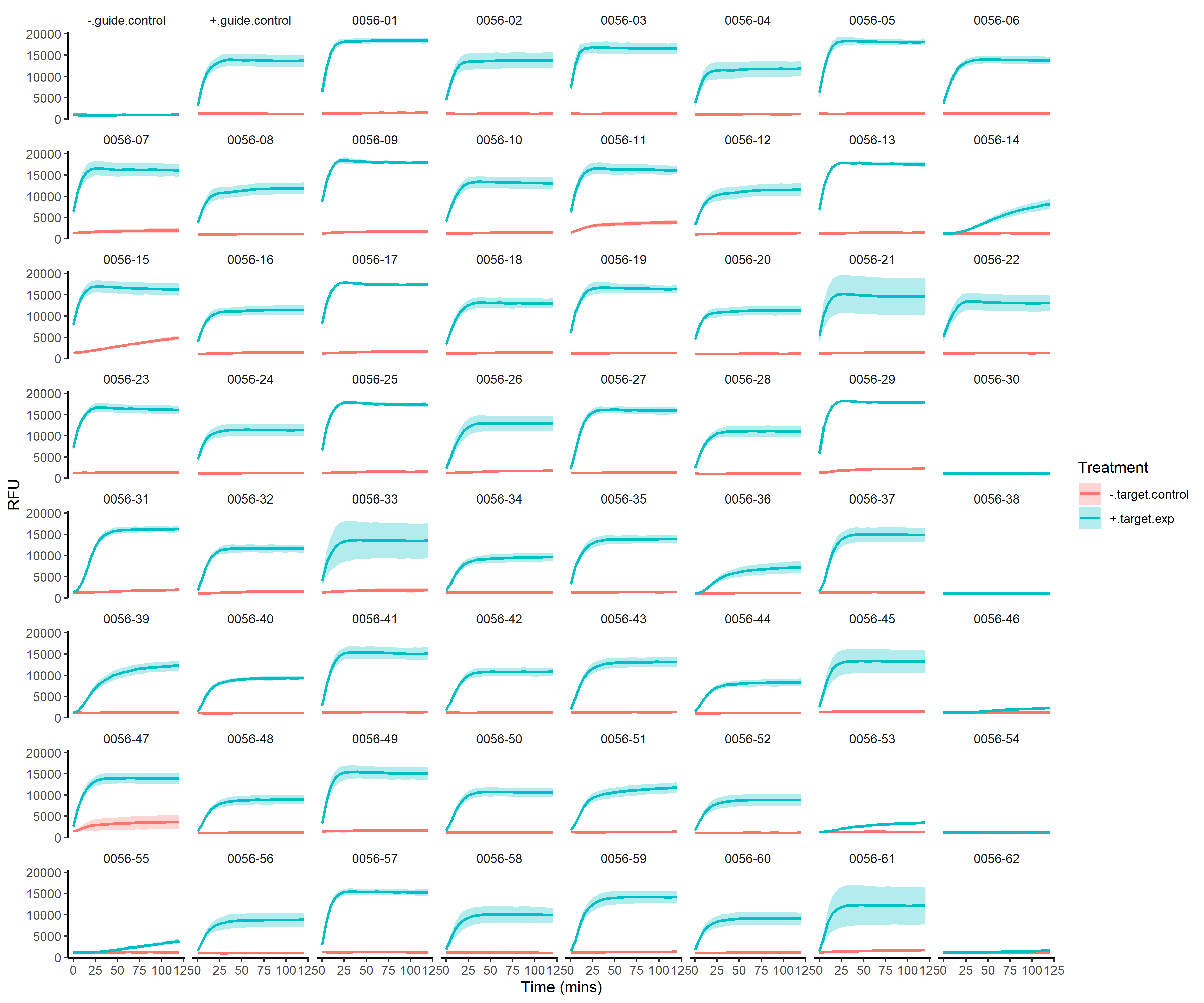


Figure S5. Fluorescence traces of guide set 0056 repeat experiment with controls of no target (red) or no guide (TE neg control) over time.


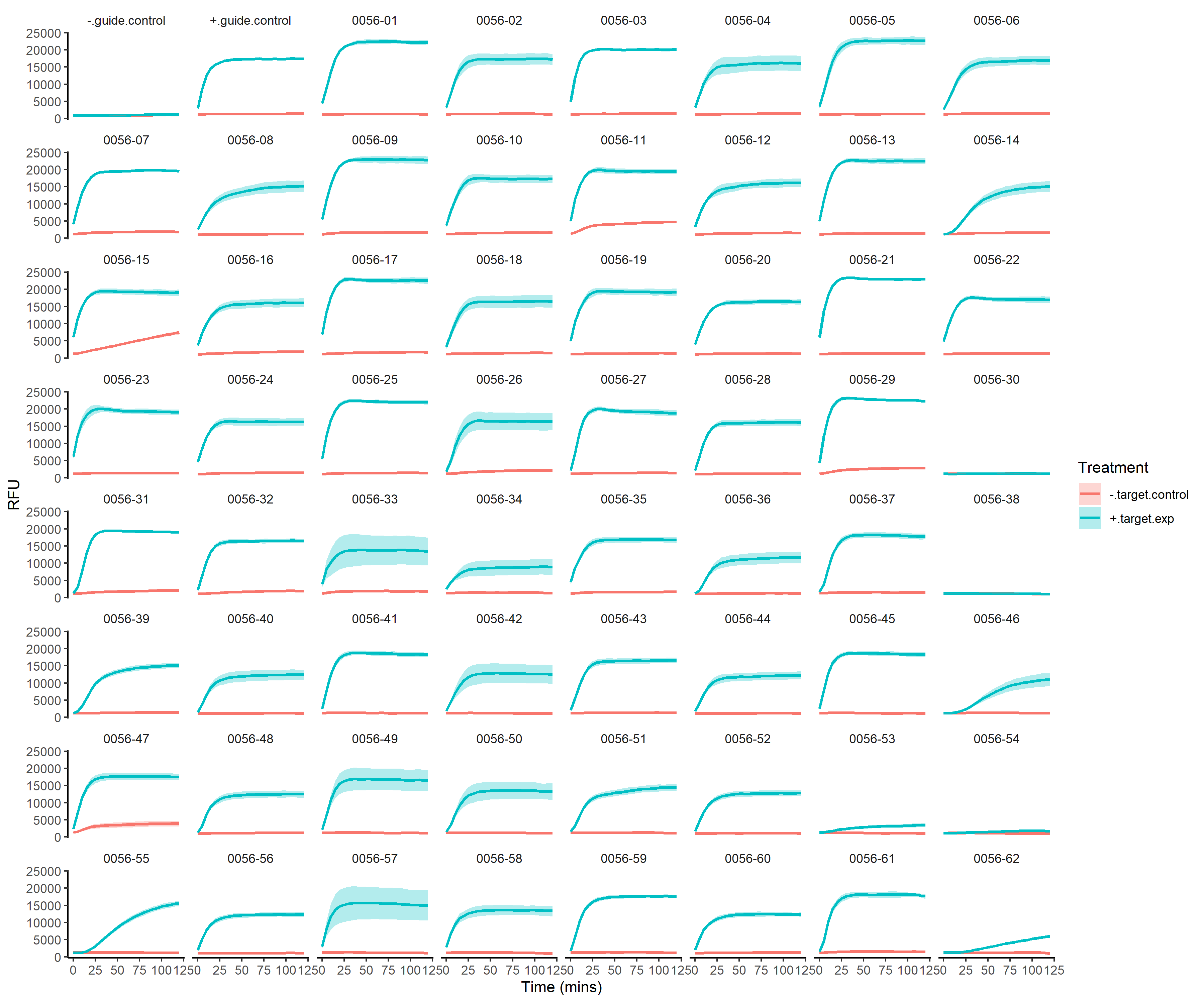


Figure S6. Fluorescence traces of guide set 0056 against shortened target with controls of no target (red) or no guide (TE neg control) over time.


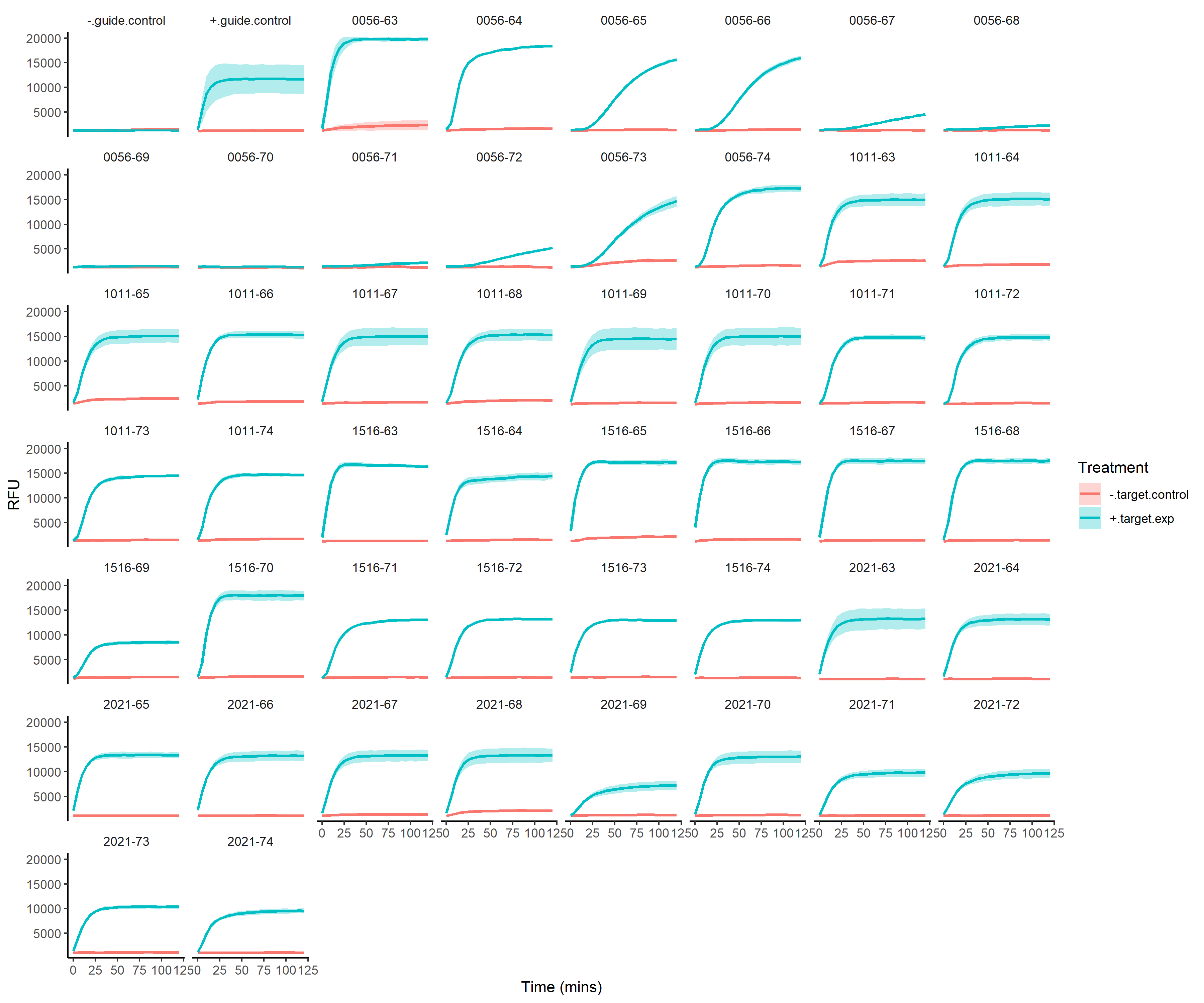


Figure S7. Fluorescence traces of remainder of guide sets with controls of no target (red) or no guide (TE neg control) over time.


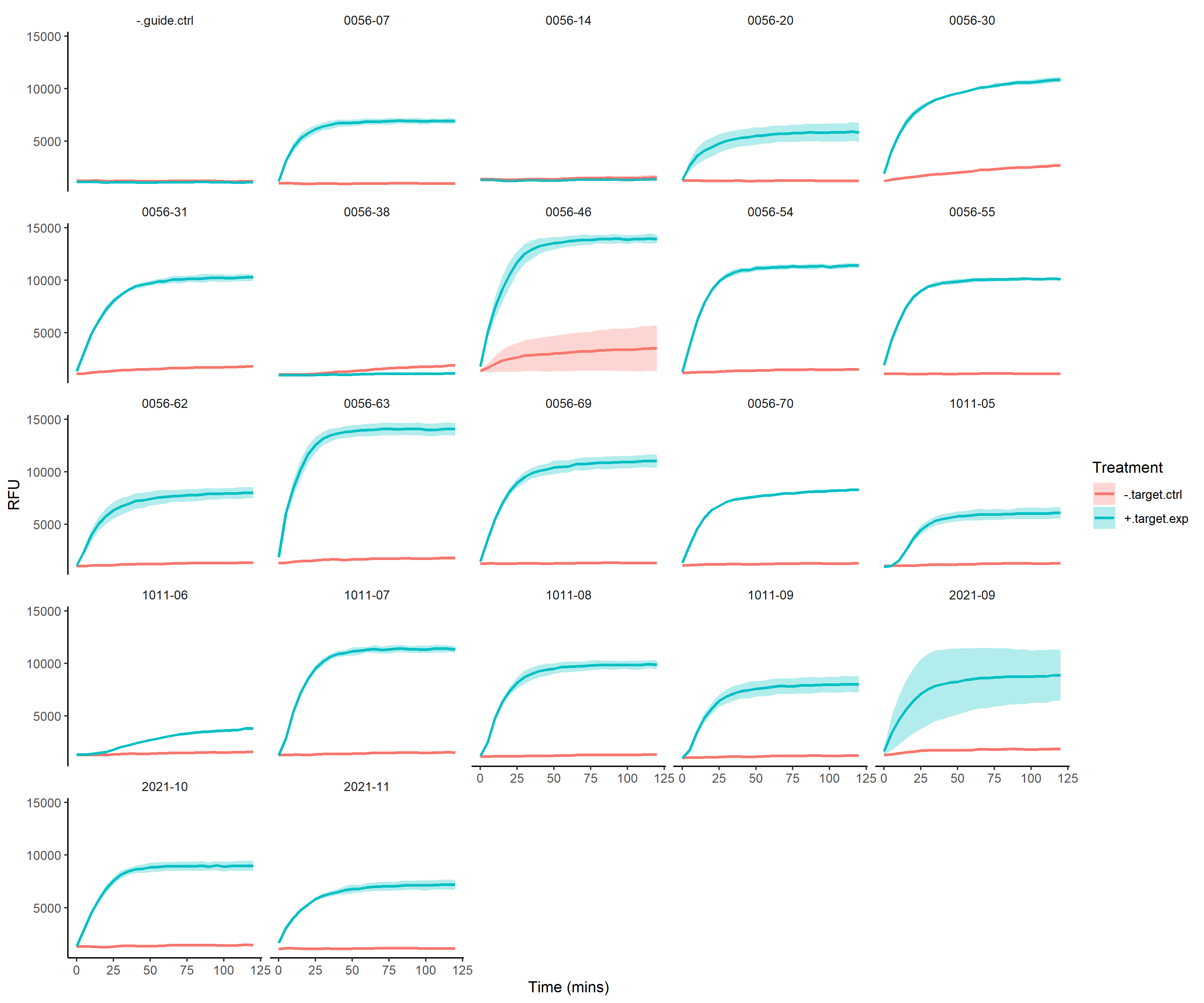


Figure S8. Fluorescence traces of select guides with DNA reordered from another company with controls of no target (red) or no guide (TE neg control) over time.

Supplemental Table 1. DNA Oligonucleotides used as templates for crRNA

| **crRNAs** | |  |  |  |  |  |  |  |  |  |  |
| --- | --- | --- | --- | --- | --- | --- | --- | --- | --- | --- | --- |
| **This Study** | **Prev. Study** | **DNA Oligonucleotide Template Sequence (5’ – 3’)** |  |  |  |  |  |  |  |  |  |
| 0056-1 |  | *GGTTCAGTTAGTCAAAGATAAAAATATA*GTTTTAGTCCCCTTCGTTTTTGGGGTAGTCTAAATCCCC**CTATAGTGAGTCGTATTAATTTC** |  |  |  |  |  |  |  |  |  |
| 0056-2 |  | *GTTCAGTTAGTCAAAGATAAAAATATAG*GTTTTAGTCCCCTTCGTTTTTGGGGTAGTCTAAATCCCC**CTATAGTGAGTCGTATTAATTTC** |  |  |  |  |  |  |  |  |  |
| 0056-3 |  | *TTCAGTTAGTCAAAGATAAAAATATAGA*GTTTTAGTCCCCTTCGTTTTTGGGGTAGTCTAAATCCCC**CTATAGTGAGTCGTATTAATTTC** |  |  |  |  |  |  |  |  |  |
| 0056-4 |  | *TCAGTTAGTCAAAGATAAAAATATAGAT*GTTTTAGTCCCCTTCGTTTTTGGGGTAGTCTAAATCCCC**CTATAGTGAGTCGTATTAATTTC** |  |  |  |  |  |  |  |  |  |
| 0056-5 |  | *CAGTTAGTCAAAGATAAAAATATAGATA*GTTTTAGTCCCCTTCGTTTTTGGGGTAGTCTAAATCCCC**CTATAGTGAGTCGTATTAATTTC** |  |  |  |  |  |  |  |  |  |
| 0056-6 |  | *AGTTAGTCAAAGATAAAAATATAGATAT*GTTTTAGTCCCCTTCGTTTTTGGGGTAGTCTAAATCCCC**CTATAGTGAGTCGTATTAATTTC** |  |  |  |  |  |  |  |  |  |
| 0056-7 |  | *GTTAGTCAAAGATAAAAATATAGATATT*GTTTTAGTCCCCTTCGTTTTTGGGGTAGTCTAAATCCCC**CTATAGTGAGTCGTATTAATTTC** |  |  |  |  |  |  |  |  |  |
| 0056-8 |  | *TTAGTCAAAGATAAAAATATAGATATTT*GTTTTAGTCCCCTTCGTTTTTGGGGTAGTCTAAATCCCC**CTATAGTGAGTCGTATTAATTTC** |  |  |  |  |  |  |  |  |  |
|  | crRNA5 | *TAGTCAAAGATAAAAATATAGATATTTC*GTTTTAGTCCCCTTCGTTTTTGGGGTAGTCTAAATCCCC**CTATAGTGAGTCGTATTAATTTC** |  |  |  |  |  |  |  |  |  |
| 0056-9 |  | *AGTCAAAGATAAAAATATAGATATTTCC*GTTTTAGTCCCCTTCGTTTTTGGGGTAGTCTAAATCCCC**CTATAGTGAGTCGTATTAATTTC** |  |  |  |  |  |  |  |  |  |
| 0056-10 |  | *GTCAAAGATAAAAATATAGATATTTCCA*GTTTTAGTCCCCTTCGTTTTTGGGGTAGTCTAAATCCCC**CTATAGTGAGTCGTATTAATTTC** |  |  |  |  |  |  |  |  |  |
| 0056-11 |  | *TCAAAGATAAAAATATAGATATTTCCAT*GTTTTAGTCCCCTTCGTTTTTGGGGTAGTCTAAATCCCC**CTATAGTGAGTCGTATTAATTTC** |  |  |  |  |  |  |  |  |  |
| 0056-12 |  | *CAAAGATAAAAATATAGATATTTCCATT*GTTTTAGTCCCCTTCGTTTTTGGGGTAGTCTAAATCCCC**CTATAGTGAGTCGTATTAATTTC** |  |  |  |  |  |  |  |  |  |
| 0056-13 |  | *AAAGATAAAAATATAGATATTTCCATTA*GTTTTAGTCCCCTTCGTTTTTGGGGTAGTCTAAATCCCC**CTATAGTGAGTCGTATTAATTTC** |  |  |  |  |  |  |  |  |  |
| 0056-14 |  | *AAGATAAAAATATAGATATTTCCATTAA*GTTTTAGTCCCCTTCGTTTTTGGGGTAGTCTAAATCCCC**CTATAGTGAGTCGTATTAATTTC** |  |  |  |  |  |  |  |  |  |
| 0056-15 |  | *AGATAAAAATATAGATATTTCCATTAAA*GTTTTAGTCCCCTTCGTTTTTGGGGTAGTCTAAATCCCC**CTATAGTGAGTCGTATTAATTTC** |  |  |  |  |  |  |  |  |  |
| 0056-16 |  | *GATAAAAATATAGATATTTCCATTAAAT*GTTTTAGTCCCCTTCGTTTTTGGGGTAGTCTAAATCCCC**CTATAGTGAGTCGTATTAATTTC** |  |  |  |  |  |  |  |  |  |
| 0056-17 |  | *ATAAAAATATAGATATTTCCATTAAATA*GTTTTAGTCCCCTTCGTTTTTGGGGTAGTCTAAATCCCC**CTATAGTGAGTCGTATTAATTTC** |  |  |  |  |  |  |  |  |  |
| 0056-18 |  | *TAAAAATATAGATATTTCCATTAAATAT*GTTTTAGTCCCCTTCGTTTTTGGGGTAGTCTAAATCCCC**CTATAGTGAGTCGTATTAATTTC** |  |  |  |  |  |  |  |  |  |
| 0056-19 |  | *AAAAATATAGATATTTCCATTAAATATG*GTTTTAGTCCCCTTCGTTTTTGGGGTAGTCTAAATCCCC**CTATAGTGAGTCGTATTAATTTC** |  |  |  |  |  |  |  |  |  |
| 0056-20 |  | *AAAATATAGATATTTCCATTAAATATGA*GTTTTAGTCCCCTTCGTTTTTGGGGTAGTCTAAATCCCC**CTATAGTGAGTCGTATTAATTTC** |  |  |  |  |  |  |  |  |  |
| 0056-21 |  | *AAATATAGATATTTCCATTAAATATGAT*GTTTTAGTCCCCTTCGTTTTTGGGGTAGTCTAAATCCCC**CTATAGTGAGTCGTATTAATTTC** |  |  |  |  |  |  |  |  |  |
| 0056-22 |  | *AATATAGATATTTCCATTAAATATGATC*GTTTTAGTCCCCTTCGTTTTTGGGGTAGTCTAAATCCCC**CTATAGTGAGTCGTATTAATTTC** |  |  |  |  |  |  |  |  |  |
| 0056-23 |  | *ATATAGATATTTCCATTAAATATGATCC*GTTTTAGTCCCCTTCGTTTTTGGGGTAGTCTAAATCCCC**CTATAGTGAGTCGTATTAATTTC** |  |  |  |  |  |  |  |  |  |
| 0056-24 |  | *TATAGATATTTCCATTAAATATGATCCC*GTTTTAGTCCCCTTCGTTTTTGGGGTAGTCTAAATCCCC**CTATAGTGAGTCGTATTAATTTC** |  |  |  |  |  |  |  |  |  |
| 0056-25 |  | *ATAGATATTTCCATTAAATATGATCCCA*GTTTTAGTCCCCTTCGTTTTTGGGGTAGTCTAAATCCCC**CTATAGTGAGTCGTATTAATTTC** |  |  |  |  |  |  |  |  |  |
| 0056-26 |  | *TAGATATTTCCATTAAATATGATCCCAG*GTTTTAGTCCCCTTCGTTTTTGGGGTAGTCTAAATCCCC**CTATAGTGAGTCGTATTAATTTC** |  |  |  |  |  |  |  |  |  |
| 0056-27 |  | *AGATATTTCCATTAAATATGATCCCAGA*GTTTTAGTCCCCTTCGTTTTTGGGGTAGTCTAAATCCCC**CTATAGTGAGTCGTATTAATTTC** |  |  |  |  |  |  |  |  |  |
| 0056-28 |  | *GATATTTCCATTAAATATGATCCCAGAA*GTTTTAGTCCCCTTCGTTTTTGGGGTAGTCTAAATCCCC**CTATAGTGAGTCGTATTAATTTC** |  |  |  |  |  |  |  |  |  |
| 0056-29 |  | *ATATTTCCATTAAATATGATCCCAGAAA*GTTTTAGTCCCCTTCGTTTTTGGGGTAGTCTAAATCCCC**CTATAGTGAGTCGTATTAATTTC** |  |  |  |  |  |  |  |  |  |
| 0056-30 |  | *TATTTCCATTAAATATGATCCCAGAAAA*GTTTTAGTCCCCTTCGTTTTTGGGGTAGTCTAAATCCCC**CTATAGTGAGTCGTATTAATTTC** |  |  |  |  |  |  |  |  |  |
| 0056-31 |  | *ATTTCCATTAAATATGATCCCAGAAAAG*GTTTTAGTCCCCTTCGTTTTTGGGGTAGTCTAAATCCCC**CTATAGTGAGTCGTATTAATTTC** |  |  |  |  |  |  |  |  |  |
| 0056-32 |  | *TTTCCATTAAATATGATCCCAGAAAAGA*GTTTTAGTCCCCTTCGTTTTTGGGGTAGTCTAAATCCCC**CTATAGTGAGTCGTATTAATTTC** |  |  |  |  |  |  |  |  |  |
| 0056-33 |  | *TTCCATTAAATATGATCCCAGAAAAGAT*GTTTTAGTCCCCTTCGTTTTTGGGGTAGTCTAAATCCCC**CTATAGTGAGTCGTATTAATTTC** |  |  |  |  |  |  |  |  |  |
|  | crRNA6 | *TCCATTAAATATGATCCCAGAAAAGATT*GTTTTAGTCCCCTTCGTTTTTGGGGTAGTCTAAATCCCC**CTATAGTGAGTCGTATTAATTTC** |  |  |  |  |  |  |  |  |  |
| 0056-34 |  | *CCATTAAATATGATCCCAGAAAAGATTC*GTTTTAGTCCCCTTCGTTTTTGGGGTAGTCTAAATCCCC**CTATAGTGAGTCGTATTAATTTC** |  |  |  |  |  |  |  |  |  |
| 0056-35 |  | *CATTAAATATGATCCCAGAAAAGATTCG*GTTTTAGTCCCCTTCGTTTTTGGGGTAGTCTAAATCCCC**CTATAGTGAGTCGTATTAATTTC** |  |  |  |  |  |  |  |  |  |
| 0056-36 |  | *ATTAAATATGATCCCAGAAAAGATTCGG*GTTTTAGTCCCCTTCGTTTTTGGGGTAGTCTAAATCCCC**CTATAGTGAGTCGTATTAATTTC** |  |  |  |  |  |  |  |  |  |
| 0056-37 |  | *TTAAATATGATCCCAGAAAAGATTCGGA*GTTTTAGTCCCCTTCGTTTTTGGGGTAGTCTAAATCCCC**CTATAGTGAGTCGTATTAATTTC** |  |  |  |  |  |  |  |  |  |
| 0056-38 |  | *TAAATATGATCCCAGAAAAGATTCGGAG*GTTTTAGTCCCCTTCGTTTTTGGGGTAGTCTAAATCCCC**CTATAGTGAGTCGTATTAATTTC** |  |  |  |  |  |  |  |  |  |
| 0056-39 |  | *AAATATGATCCCAGAAAAGATTCGGAGG*GTTTTAGTCCCCTTCGTTTTTGGGGTAGTCTAAATCCCC**CTATAGTGAGTCGTATTAATTTC** |  |  |  |  |  |  |  |  |  |
| 0056-40 |  | *AATATGATCCCAGAAAAGATTCGGAGGT*GTTTTAGTCCCCTTCGTTTTTGGGGTAGTCTAAATCCCC**CTATAGTGAGTCGTATTAATTTC** |  |  |  |  |  |  |  |  |  |
| 0056-41 |  | *ATATGATCCCAGAAAAGATTCGGAGGTT*GTTTTAGTCCCCTTCGTTTTTGGGGTAGTCTAAATCCCC**CTATAGTGAGTCGTATTAATTTC** |  |  |  |  |  |  |  |  |  |
| 0056-42 |  | *TATGATCCCAGAAAAGATTCGGAGGTTT*GTTTTAGTCCCCTTCGTTTTTGGGGTAGTCTAAATCCCC**CTATAGTGAGTCGTATTAATTTC** |  |  |  |  |  |  |  |  |  |
| 0056-43 |  | *ATGATCCCAGAAAAGATTCGGAGGTTTT*GTTTTAGTCCCCTTCGTTTTTGGGGTAGTCTAAATCCCC**CTATAGTGAGTCGTATTAATTTC** |  |  |  |  |  |  |  |  |  |
| 0056-44 |  | *TGATCCCAGAAAAGATTCGGAGGTTTTT*GTTTTAGTCCCCTTCGTTTTTGGGGTAGTCTAAATCCCC**CTATAGTGAGTCGTATTAATTTC** |  |  |  |  |  |  |  |  |  |
| 0056-45 |  | *GATCCCAGAAAAGATTCGGAGGTTTTTG*GTTTTAGTCCCCTTCGTTTTTGGGGTAGTCTAAATCCCC**CTATAGTGAGTCGTATTAATTTC** |  |  |  |  |  |  |  |  |  |
| 0056-46 |  | *ATCCCAGAAAAGATTCGGAGGTTTTTGC*GTTTTAGTCCCCTTCGTTTTTGGGGTAGTCTAAATCCCC**CTATAGTGAGTCGTATTAATTTC** |  |  |  |  |  |  |  |  |  |
| 0056-47 |  | *TCCCAGAAAAGATTCGGAGGTTTTTGCC*GTTTTAGTCCCCTTCGTTTTTGGGGTAGTCTAAATCCCC**CTATAGTGAGTCGTATTAATTTC** |  |  |  |  |  |  |  |  |  |
| 0056-48 |  | *CCCAGAAAAGATTCGGAGGTTTTTGCCA*GTTTTAGTCCCCTTCGTTTTTGGGGTAGTCTAAATCCCC**CTATAGTGAGTCGTATTAATTTC** |  |  |  |  |  |  |  |  |  |
| 0056-49 |  | *CCAGAAAAGATTCGGAGGTTTTTGCCAA*GTTTTAGTCCCCTTCGTTTTTGGGGTAGTCTAAATCCCC**CTATAGTGAGTCGTATTAATTTC** |  |  |  |  |  |  |  |  |  |
| 0056-50 |  | *CAGAAAAGATTCGGAGGTTTTTGCCAAT*GTTTTAGTCCCCTTCGTTTTTGGGGTAGTCTAAATCCCC**CTATAGTGAGTCGTATTAATTTC** |  |  |  |  |  |  |  |  |  |
| 0056-51 |  | *AGAAAAGATTCGGAGGTTTTTGCCAATA*GTTTTAGTCCCCTTCGTTTTTGGGGTAGTCTAAATCCCC**CTATAGTGAGTCGTATTAATTTC** |  |  |  |  |  |  |  |  |  |
| 0056-52 |  | *GAAAAGATTCGGAGGTTTTTGCCAATAG*GTTTTAGTCCCCTTCGTTTTTGGGGTAGTCTAAATCCCC**CTATAGTGAGTCGTATTAATTTC** |  |  |  |  |  |  |  |  |  |
| 0056-53 |  | *AAAAGATTCGGAGGTTTTTGCCAATAGA*GTTTTAGTCCCCTTCGTTTTTGGGGTAGTCTAAATCCCC**CTATAGTGAGTCGTATTAATTTC** |  |  |  |  |  |  |  |  |  |
| 0056-54 |  | *AAAGATTCGGAGGTTTTTGCCAATAGAG*GTTTTAGTCCCCTTCGTTTTTGGGGTAGTCTAAATCCCC**CTATAGTGAGTCGTATTAATTTC** |  |  |  |  |  |  |  |  |  |
| 0056-55 |  | *AAGATTCGGAGGTTTTTGCCAATAGAGT*GTTTTAGTCCCCTTCGTTTTTGGGGTAGTCTAAATCCCC**CTATAGTGAGTCGTATTAATTTC** |  |  |  |  |  |  |  |  |  |
| 0056-56 |  | *AGATTCGGAGGTTTTTGCCAATAGAGTA*GTTTTAGTCCCCTTCGTTTTTGGGGTAGTCTAAATCCCC**CTATAGTGAGTCGTATTAATTTC** |  |  |  |  |  |  |  |  |  |
| 0056-57 |  | *GATTCGGAGGTTTTTGCCAATAGAGTAA*GTTTTAGTCCCCTTCGTTTTTGGGGTAGTCTAAATCCCC**CTATAGTGAGTCGTATTAATTTC** |  |  |  |  |  |  |  |  |  |
| 0056-58 |  | *ATTCGGAGGTTTTTGCCAATAGAGTAAT*GTTTTAGTCCCCTTCGTTTTTGGGGTAGTCTAAATCCCC**CTATAGTGAGTCGTATTAATTTC** |  |  |  |  |  |  |  |  |  |
| 0056-59 |  | *TCGGAGGTTTTTGCCAATAGAGTAATTA*GTTTTAGTCCCCTTCGTTTTTGGGGTAGTCTAAATCCCC**CTATAGTGAGTCGTATTAATTTC** |  |  |  |  |  |  |  |  |  |
| 0056-60 |  | *CGGAGGTTTTTGCCAATAGAGTAATTAC*GTTTTAGTCCCCTTCGTTTTTGGGGTAGTCTAAATCCCC**CTATAGTGAGTCGTATTAATTTC** |  |  |  |  |  |  |  |  |  |
| 0056-61 |  | *GGAGGTTTTTGCCAATAGAGTAATTACT*GTTTTAGTCCCCTTCGTTTTTGGGGTAGTCTAAATCCCC**CTATAGTGAGTCGTATTAATTTC** |  |  |  |  |  |  |  |  |  |
| 0056-62 |  | *GAGGTTTTTGCCAATAGAGTAATTACTG*GTTTTAGTCCCCTTCGTTTTTGGGGTAGTCTAAATCCCC**CTATAGTGAGTCGTATTAATTTC** |  |  |  |  |  |  |  |  |  |
| 0056-63 |  | *AGGTTTTTGCCAATAGAGTAATTACTGA*GTTTTAGTCCCCTTCGTTTTTGGGGTAGTCTAAATCCCC**CTATAGTGAGTCGTATTAATTTC** |  |  |  |  |  |  |  |  |  |
| 0056-64 |  | *GGTTTTTGCCAATAGAGTAATTACTGAT*GTTTTAGTCCCCTTCGTTTTTGGGGTAGTCTAAATCCCC**CTATAGTGAGTCGTATTAATTTC** |  |  |  |  |  |  |  |  |  |
| 0056-65 |  | *GTTTTTGCCAATAGAGTAATTACTGATG*GTTTTAGTCCCCTTCGTTTTTGGGGTAGTCTAAATCCCC**CTATAGTGAGTCGTATTAATTTC** |  |  |  |  |  |  |  |  |  |
| 0056-66 | crRNA7 | *TTTTTGCCAATAGAGTAATTACTGATGA*GTTTTAGTCCCCTTCGTTTTTGGGGTAGTCTAAATCCCC**CTATAGTGAGTCGTATTAATTTC** |  |  |  |  |  |  |  |  |  |
| 0056-67 |  | *TTTTGCCAATAGAGTAATTACTGATGAT*GTTTTAGTCCCCTTCGTTTTTGGGGTAGTCTAAATCCCC**CTATAGTGAGTCGTATTAATTTC** |  |  |  |  |  |  |  |  |  |
| 0056-68 |  | *TTTGCCAATAGAGTAATTACTGATGATA*GTTTTAGTCCCCTTCGTTTTTGGGGTAGTCTAAATCCCC**CTATAGTGAGTCGTATTAATTTC** |  |  |  |  |  |  |  |  |  |
| 0056-69 |  | *TTGCCAATAGAGTAATTACTGATGATAT*GTTTTAGTCCCCTTCGTTTTTGGGGTAGTCTAAATCCCC**CTATAGTGAGTCGTATTAATTTC** |  |  |  |  |  |  |  |  |  |
| 0056-70 |  | *TGCCAATAGAGTAATTACTGATGATATC*GTTTTAGTCCCCTTCGTTTTTGGGGTAGTCTAAATCCCC**CTATAGTGAGTCGTATTAATTTC** |  |  |  |  |  |  |  |  |  |
| 0056-71 |  | *GCCAATAGAGTAATTACTGATGATATCG*GTTTTAGTCCCCTTCGTTTTTGGGGTAGTCTAAATCCCC**CTATAGTGAGTCGTATTAATTTC** |  |  |  |  |  |  |  |  |  |
| 0056-72 |  | *CCAATAGAGTAATTACTGATGATATCGA*GTTTTAGTCCCCTTCGTTTTTGGGGTAGTCTAAATCCCC**CTATAGTGAGTCGTATTAATTTC** |  |  |  |  |  |  |  |  |  |
| 0056-73 |  | *CAATAGAGTAATTACTGATGATATCGAA*GTTTTAGTCCCCTTCGTTTTTGGGGTAGTCTAAATCCCC**CTATAGTGAGTCGTATTAATTTC** |  |  |  |  |  |  |  |  |  |
| 0056-74 |  | *AATAGAGTAATTACTGATGATATCGAAT*GTTTTAGTCCCCTTCGTTTTTGGGGTAGTCTAAATCCCC**CTATAGTGAGTCGTATTAATTTC** |  |  |  |  |  |  |  |  |  |
| 1011-1 |  | *ATTTTCTACCCGAGGATGCCATTCTTAA*GTTTTAGTCCCCTTCGTTTTTGGGGTAGTCTAAATCCCCCTATAGTGAGTCGTATTAATTTC |  |  |  |  |  |  |  |  |  |
| 1011-2 |  | *TTTTCTACCCGAGGATGCCATTCTTAAA*GTTTTAGTCCCCTTCGTTTTTGGGGTAGTCTAAATCCCCCTATAGTGAGTCGTATTAATTTC |  |  |  |  |  |  |  |  |  |
| 1011-3 |  | *TTTCTACCCGAGGATGCCATTCTTAAAG*GTTTTAGTCCCCTTCGTTTTTGGGGTAGTCTAAATCCCCCTATAGTGAGTCGTATTAATTTC |  |  |  |  |  |  |  |  |  |
| 1011-4 |  | *TTCTACCCGAGGATGCCATTCTTAAAGG*GTTTTAGTCCCCTTCGTTTTTGGGGTAGTCTAAATCCCCCTATAGTGAGTCGTATTAATTTC |  |  |  |  |  |  |  |  |  |
| 1011-5 |  | *TCTACCCGAGGATGCCATTCTTAAAGGC*GTTTTAGTCCCCTTCGTTTTTGGGGTAGTCTAAATCCCCCTATAGTGAGTCGTATTAATTTC |  |  |  |  |  |  |  |  |  |
| 1011-6 |  | *CTACCCGAGGATGCCATTCTTAAAGGCG*GTTTTAGTCCCCTTCGTTTTTGGGGTAGTCTAAATCCCC**CTATAGTGAGTCGTATTAATTTC** |  |  |  |  |  |  |  |  |  |
| 1011-7 |  | *TACCCGAGGATGCCATTCTTAAAGGCGG*GTTTTAGTCCCCTTCGTTTTTGGGGTAGTCTAAATCCCC**CTATAGTGAGTCGTATTAATTTC** |  |  |  |  |  |  |  |  |  |
| 1011-8 |  | *ACCCGAGGATGCCATTCTTAAAGGCGGT*GTTTTAGTCCCCTTCGTTTTTGGGGTAGTCTAAATCCCC**CTATAGTGAGTCGTATTAATTTC** |  |  |  |  |  |  |  |  |  |
| 1011-9 |  | *CCCGAGGATGCCATTCTTAAAGGCGGTC*GTTTTAGTCCCCTTCGTTTTTGGGGTAGTCTAAATCCCC**CTATAGTGAGTCGTATTAATTTC** |  |  |  |  |  |  |  |  |  |
| 1011-10 |  | *CCGAGGATGCCATTCTTAAAGGCGGTCA*GTTTTAGTCCCCTTCGTTTTTGGGGTAGTCTAAATCCCC**CTATAGTGAGTCGTATTAATTTC** |  |  |  |  |  |  |  |  |  |
| 1011-11 |  | *CGAGGATGCCATTCTTAAAGGCGGTCAT*GTTTTAGTCCCCTTCGTTTTTGGGGTAGTCTAAATCCCC**CTATAGTGAGTCGTATTAATTTC** |  |  |  |  |  |  |  |  |  |
| 1011-12 |  | *GAGGATGCCATTCTTAAAGGCGGTCATT*GTTTTAGTCCCCTTCGTTTTTGGGGTAGTCTAAATCCCC**CTATAGTGAGTCGTATTAATTTC** |  |  |  |  |  |  |  |  |  |
| 1011-13 |  | *AGGATGCCATTCTTAAAGGCGGTCATTA*GTTTTAGTCCCCTTCGTTTTTGGGGTAGTCTAAATCCCC**CTATAGTGAGTCGTATTAATTTC** |  |  |  |  |  |  |  |  |  |
| 1011-14 |  | *GGATGCCATTCTTAAAGGCGGTCATTAT*GTTTTAGTCCCCTTCGTTTTTGGGGTAGTCTAAATCCCC**CTATAGTGAGTCGTATTAATTTC** |  |  |  |  |  |  |  |  |  |
| 1011-15 |  | *GATGCCATTCTTAAAGGCGGTCATTATG*GTTTTAGTCCCCTTCGTTTTTGGGGTAGTCTAAATCCCC**CTATAGTGAGTCGTATTAATTTC** |  |  |  |  |  |  |  |  |  |
| 1011-16 |  | *ATGCCATTCTTAAAGGCGGTCATTATGA*GTTTTAGTCCCCTTCGTTTTTGGGGTAGTCTAAATCCCC**CTATAGTGAGTCGTATTAATTTC** |  |  |  |  |  |  |  |  |  |
| 1011-17 |  | *TGCCATTCTTAAAGGCGGTCATTATGAC*GTTTTAGTCCCCTTCGTTTTTGGGGTAGTCTAAATCCCC**CTATAGTGAGTCGTATTAATTTC** |  |  |  |  |  |  |  |  |  |
| 1011-18 |  | *GCCATTCTTAAAGGCGGTCATTATGACA*GTTTTAGTCCCCTTCGTTTTTGGGGTAGTCTAAATCCCC**CTATAGTGAGTCGTATTAATTTC** |  |  |  |  |  |  |  |  |  |
| 1011-19 |  | *CCATTCTTAAAGGCGGTCATTATGACAA*GTTTTAGTCCCCTTCGTTTTTGGGGTAGTCTAAATCCCC**CTATAGTGAGTCGTATTAATTTC** |  |  |  |  |  |  |  |  |  |
| 1011-20 |  | *CATTCTTAAAGGCGGTCATTATGACAAC*GTTTTAGTCCCCTTCGTTTTTGGGGTAGTCTAAATCCCC**CTATAGTGAGTCGTATTAATTTC** |  |  |  |  |  |  |  |  |  |
| 1011-21 |  | *ATTCTTAAAGGCGGTCATTATGACAACC*GTTTTAGTCCCCTTCGTTTTTGGGGTAGTCTAAATCCCC**CTATAGTGAGTCGTATTAATTTC** |  |  |  |  |  |  |  |  |  |
| 1011-22 |  | *TTCTTAAAGGCGGTCATTATGACAACCA*GTTTTAGTCCCCTTCGTTTTTGGGGTAGTCTAAATCCCC**CTATAGTGAGTCGTATTAATTTC** |  |  |  |  |  |  |  |  |  |
| 1011-23 |  | *TCTTAAAGGCGGTCATTATGACAACCAA*GTTTTAGTCCCCTTCGTTTTTGGGGTAGTCTAAATCCCC**CTATAGTGAGTCGTATTAATTTC** |  |  |  |  |  |  |  |  |  |
|  | crRNA10 | *CTTAAAGGCGGTCATTATGACAACCAAC*GTTTTAGTCCCCTTCGTTTTTGGGGTAGTCTAAATCCCC**CTATAGTGAGTCGTATTAATTTC** |  |  |  |  |  |  |  |  |  |
| 1011-24 |  | *TTAAAGGCGGTCATTATGACAACCAACT*GTTTTAGTCCCCTTCGTTTTTGGGGTAGTCTAAATCCCC**CTATAGTGAGTCGTATTAATTTC** |  |  |  |  |  |  |  |  |  |
| 1011-25 |  | *TAAAGGCGGTCATTATGACAACCAACTG*GTTTTAGTCCCCTTCGTTTTTGGGGTAGTCTAAATCCCC**CTATAGTGAGTCGTATTAATTTC** |  |  |  |  |  |  |  |  |  |
| 1011-26 |  | *AAAGGCGGTCATTATGACAACCAACTGC*GTTTTAGTCCCCTTCGTTTTTGGGGTAGTCTAAATCCCC**CTATAGTGAGTCGTATTAATTTC** |  |  |  |  |  |  |  |  |  |
| 1011-27 |  | *AAGGCGGTCATTATGACAACCAACTGCA*GTTTTAGTCCCCTTCGTTTTTGGGGTAGTCTAAATCCCC**CTATAGTGAGTCGTATTAATTTC** |  |  |  |  |  |  |  |  |  |
| 1011-28 |  | *AGGCGGTCATTATGACAACCAACTGCAA*GTTTTAGTCCCCTTCGTTTTTGGGGTAGTCTAAATCCCC**CTATAGTGAGTCGTATTAATTTC** |  |  |  |  |  |  |  |  |  |
| 1011-29 |  | *GGCGGTCATTATGACAACCAACTGCAAA*GTTTTAGTCCCCTTCGTTTTTGGGGTAGTCTAAATCCCC**CTATAGTGAGTCGTATTAATTTC** |  |  |  |  |  |  |  |  |  |
| 1011-30 |  | *GCGGTCATTATGACAACCAACTGCAAAA*GTTTTAGTCCCCTTCGTTTTTGGGGTAGTCTAAATCCCC**CTATAGTGAGTCGTATTAATTTC** |  |  |  |  |  |  |  |  |  |
| 1011-31 |  | *CGGTCATTATGACAACCAACTGCAAAAT*GTTTTAGTCCCCTTCGTTTTTGGGGTAGTCTAAATCCCC**CTATAGTGAGTCGTATTAATTTC** |  |  |  |  |  |  |  |  |  |
| 1011-32 |  | *GGTCATTATGACAACCAACTGCAAAATG*GTTTTAGTCCCCTTCGTTTTTGGGGTAGTCTAAATCCCC**CTATAGTGAGTCGTATTAATTTC** |  |  |  |  |  |  |  |  |  |
| 1011-33 |  | *GTCATTATGACAACCAACTGCAAAATGG*GTTTTAGTCCCCTTCGTTTTTGGGGTAGTCTAAATCCCC**CTATAGTGAGTCGTATTAATTTC** |  |  |  |  |  |  |  |  |  |
| 1011-34 |  | *TCATTATGACAACCAACTGCAAAATGGC*GTTTTAGTCCCCTTCGTTTTTGGGGTAGTCTAAATCCCC**CTATAGTGAGTCGTATTAATTTC** |  |  |  |  |  |  |  |  |  |
| 1011-35 |  | *CATTATGACAACCAACTGCAAAATGGCA*GTTTTAGTCCCCTTCGTTTTTGGGGTAGTCTAAATCCCC**CTATAGTGAGTCGTATTAATTTC** |  |  |  |  |  |  |  |  |  |
| 1011-36 |  | *ATTATGACAACCAACTGCAAAATGGCAT*GTTTTAGTCCCCTTCGTTTTTGGGGTAGTCTAAATCCCC**CTATAGTGAGTCGTATTAATTTC** |  |  |  |  |  |  |  |  |  |
| 1011-37 |  | *TTATGACAACCAACTGCAAAATGGCATC*GTTTTAGTCCCCTTCGTTTTTGGGGTAGTCTAAATCCCC**CTATAGTGAGTCGTATTAATTTC** |  |  |  |  |  |  |  |  |  |
| 1011-38 |  | *TATGACAACCAACTGCAAAATGGCATCA*GTTTTAGTCCCCTTCGTTTTTGGGGTAGTCTAAATCCCC**CTATAGTGAGTCGTATTAATTTC** |  |  |  |  |  |  |  |  |  |
| 1011-39 |  | *ATGACAACCAACTGCAAAATGGCATCAA*GTTTTAGTCCCCTTCGTTTTTGGGGTAGTCTAAATCCCC**CTATAGTGAGTCGTATTAATTTC** |  |  |  |  |  |  |  |  |  |
| 1011-40 |  | *TGACAACCAACTGCAAAATGGCATCAAG*GTTTTAGTCCCCTTCGTTTTTGGGGTAGTCTAAATCCCC**CTATAGTGAGTCGTATTAATTTC** |  |  |  |  |  |  |  |  |  |
| 1011-41 |  | *GACAACCAACTGCAAAATGGCATCAAGC*GTTTTAGTCCCCTTCGTTTTTGGGGTAGTCTAAATCCCC**CTATAGTGAGTCGTATTAATTTC** |  |  |  |  |  |  |  |  |  |
| 1011-42 |  | *ACAACCAACTGCAAAATGGCATCAAGCG*GTTTTAGTCCCCTTCGTTTTTGGGGTAGTCTAAATCCCC**CTATAGTGAGTCGTATTAATTTC** |  |  |  |  |  |  |  |  |  |
| 1011-43 |  | *CAACCAACTGCAAAATGGCATCAAGCGA*GTTTTAGTCCCCTTCGTTTTTGGGGTAGTCTAAATCCCC**CTATAGTGAGTCGTATTAATTTC** |  |  |  |  |  |  |  |  |  |
| 1011-44 |  | *AACCAACTGCAAAATGGCATCAAGCGAG*GTTTTAGTCCCCTTCGTTTTTGGGGTAGTCTAAATCCCC**CTATAGTGAGTCGTATTAATTTC** |  |  |  |  |  |  |  |  |  |
| 1011-45 |  | *ACCAACTGCAAAATGGCATCAAGCGAGT*GTTTTAGTCCCCTTCGTTTTTGGGGTAGTCTAAATCCCC**CTATAGTGAGTCGTATTAATTTC** |  |  |  |  |  |  |  |  |  |
| 1011-46 |  | *CCAACTGCAAAATGGCATCAAGCGAGTA*GTTTTAGTCCCCTTCGTTTTTGGGGTAGTCTAAATCCCC**CTATAGTGAGTCGTATTAATTTC** |  |  |  |  |  |  |  |  |  |
| 1011-47 |  | *CAACTGCAAAATGGCATCAAGCGAGTAA*GTTTTAGTCCCCTTCGTTTTTGGGGTAGTCTAAATCCCC**CTATAGTGAGTCGTATTAATTTC** |  |  |  |  |  |  |  |  |  |
| 1011-48 |  | *AACTGCAAAATGGCATCAAGCGAGTAAA*GTTTTAGTCCCCTTCGTTTTTGGGGTAGTCTAAATCCCC**CTATAGTGAGTCGTATTAATTTC** |  |  |  |  |  |  |  |  |  |
| 1011-49 |  | *ACTGCAAAATGGCATCAAGCGAGTAAAA*GTTTTAGTCCCCTTCGTTTTTGGGGTAGTCTAAATCCCC**CTATAGTGAGTCGTATTAATTTC** |  |  |  |  |  |  |  |  |  |
| 1011-50 |  | *CTGCAAAATGGCATCAAGCGAGTAAAAG*GTTTTAGTCCCCTTCGTTTTTGGGGTAGTCTAAATCCCC**CTATAGTGAGTCGTATTAATTTC** |  |  |  |  |  |  |  |  |  |
|  | crRNA11 | *TGCAAAATGGCATCAAGCGAGTAAAAGA*GTTTTAGTCCCCTTCGTTTTTGGGGTAGTCTAAATCCCC**CTATAGTGAGTCGTATTAATTTC** |  |  |  |  |  |  |  |  |  |
| 1011-51 |  | *GCAAAATGGCATCAAGCGAGTAAAAGAG*GTTTTAGTCCCCTTCGTTTTTGGGGTAGTCTAAATCCCC**CTATAGTGAGTCGTATTAATTTC** |  |  |  |  |  |  |  |  |  |
| 1011-52 |  | *CAAAATGGCATCAAGCGAGTAAAAGAGT*GTTTTAGTCCCCTTCGTTTTTGGGGTAGTCTAAATCCCC**CTATAGTGAGTCGTATTAATTTC** |  |  |  |  |  |  |  |  |  |
| 1011-53 |  | *AAAATGGCATCAAGCGAGTAAAAGAGTT*GTTTTAGTCCCCTTCGTTTTTGGGGTAGTCTAAATCCCC**CTATAGTGAGTCGTATTAATTTC** |  |  |  |  |  |  |  |  |  |
| 1011-54 |  | *AAATGGCATCAAGCGAGTAAAAGAGTTC*GTTTTAGTCCCCTTCGTTTTTGGGGTAGTCTAAATCCCC**CTATAGTGAGTCGTATTAATTTC** |  |  |  |  |  |  |  |  |  |
| 1011-55 |  | *AATGGCATCAAGCGAGTAAAAGAGTTCC*GTTTTAGTCCCCTTCGTTTTTGGGGTAGTCTAAATCCCC**CTATAGTGAGTCGTATTAATTTC** |  |  |  |  |  |  |  |  |  |
| 1011-56 |  | *ATGGCATCAAGCGAGTAAAAGAGTTCCT*GTTTTAGTCCCCTTCGTTTTTGGGGTAGTCTAAATCCCC**CTATAGTGAGTCGTATTAATTTC** |  |  |  |  |  |  |  |  |  |
| 1011-57 |  | *TGGCATCAAGCGAGTAAAAGAGTTCCTT*GTTTTAGTCCCCTTCGTTTTTGGGGTAGTCTAAATCCCC**CTATAGTGAGTCGTATTAATTTC** |  |  |  |  |  |  |  |  |  |
| 1011-58 |  | *GGCATCAAGCGAGTAAAAGAGTTCCTTG*GTTTTAGTCCCCTTCGTTTTTGGGGTAGTCTAAATCCCC**CTATAGTGAGTCGTATTAATTTC** |  |  |  |  |  |  |  |  |  |
| 1011-59 |  | *GCATCAAGCGAGTAAAAGAGTTCCTTGA*GTTTTAGTCCCCTTCGTTTTTGGGGTAGTCTAAATCCCC**CTATAGTGAGTCGTATTAATTTC** |  |  |  |  |  |  |  |  |  |
| 1011-60 |  | *CATCAAGCGAGTAAAAGAGTTCCTTGAA*GTTTTAGTCCCCTTCGTTTTTGGGGTAGTCTAAATCCCC**CTATAGTGAGTCGTATTAATTTC** |  |  |  |  |  |  |  |  |  |
| 1011-61 |  | *ATCAAGCGAGTAAAAGAGTTCCTTGAAT*GTTTTAGTCCCCTTCGTTTTTGGGGTAGTCTAAATCCCC**CTATAGTGAGTCGTATTAATTTC** |  |  |  |  |  |  |  |  |  |
| 1011-62 |  | *TCAAGCGAGTAAAAGAGTTCCTTGAATC*GTTTTAGTCCCCTTCGTTTTTGGGGTAGTCTAAATCCCC**CTATAGTGAGTCGTATTAATTTC** |  |  |  |  |  |  |  |  |  |
| 1011-63 |  | *CAAGCGAGTAAAAGAGTTCCTTGAATCA*GTTTTAGTCCCCTTCGTTTTTGGGGTAGTCTAAATCCCC**CTATAGTGAGTCGTATTAATTTC** |  |  |  |  |  |  |  |  |  |
| 1011-64 |  | *AAGCGAGTAAAAGAGTTCCTTGAATCAT*GTTTTAGTCCCCTTCGTTTTTGGGGTAGTCTAAATCCCC**CTATAGTGAGTCGTATTAATTTC** |  |  |  |  |  |  |  |  |  |
| 1011-65 |  | *AGCGAGTAAAAGAGTTCCTTGAATCATC*GTTTTAGTCCCCTTCGTTTTTGGGGTAGTCTAAATCCCC**CTATAGTGAGTCGTATTAATTTC** |  |  |  |  |  |  |  |  |  |
| 1011-66 |  | *GCGAGTAAAAGAGTTCCTTGAATCATCG*GTTTTAGTCCCCTTCGTTTTTGGGGTAGTCTAAATCCCC**CTATAGTGAGTCGTATTAATTTC** |  |  |  |  |  |  |  |  |  |
| 1011-67 |  | *CGAGTAAAAGAGTTCCTTGAATCATCGC*GTTTTAGTCCCCTTCGTTTTTGGGGTAGTCTAAATCCCC**CTATAGTGAGTCGTATTAATTTC** |  |  |  |  |  |  |  |  |  |
| 1011-68 |  | *GAGTAAAAGAGTTCCTTGAATCATCGCC*GTTTTAGTCCCCTTCGTTTTTGGGGTAGTCTAAATCCCC**CTATAGTGAGTCGTATTAATTTC** |  |  |  |  |  |  |  |  |  |
| 1011-69 |  | *AGTAAAAGAGTTCCTTGAATCATCGCCG*GTTTTAGTCCCCTTCGTTTTTGGGGTAGTCTAAATCCCC**CTATAGTGAGTCGTATTAATTTC** |  |  |  |  |  |  |  |  |  |
| 1011-70 |  | *GTAAAAGAGTTCCTTGAATCATCGCCGA*GTTTTAGTCCCCTTCGTTTTTGGGGTAGTCTAAATCCCC**CTATAGTGAGTCGTATTAATTTC** |  |  |  |  |  |  |  |  |  |
| 1011-71 |  | *TAAAAGAGTTCCTTGAATCATCGCCGAA*GTTTTAGTCCCCTTCGTTTTTGGGGTAGTCTAAATCCCC**CTATAGTGAGTCGTATTAATTTC** |  |  |  |  |  |  |  |  |  |
| 1011-72 |  | *AAAAGAGTTCCTTGAATCATCGCCGAAT*GTTTTAGTCCCCTTCGTTTTTGGGGTAGTCTAAATCCCC**CTATAGTGAGTCGTATTAATTTC** |  |  |  |  |  |  |  |  |  |
| 1011-73 |  | *AAAGAGTTCCTTGAATCATCGCCGAATA*GTTTTAGTCCCCTTCGTTTTTGGGGTAGTCTAAATCCCC**CTATAGTGAGTCGTATTAATTTC** |  |  |  |  |  |  |  |  |  |
| 1011-74 |  | *AAGAGTTCCTTGAATCATCGCCGAATAC*GTTTTAGTCCCCTTCGTTTTTGGGGTAGTCTAAATCCCC**CTATAGTGAGTCGTATTAATTTC** |  |  |  |  |  |  |  |  |  |
| 1516-1 |  | *ATTTCTCTTTAACCGCCGATCGTATCGA*GTTTTAGTCCCCTTCGTTTTTGGGGTAGTCTAAATCCCC**CTATAGTGAGTCGTATTAATTTC** |  |  |  |  |  |  |  |  |  |
| 1516-2 |  | *TTTCTCTTTAACCGCCGATCGTATCGAT*GTTTTAGTCCCCTTCGTTTTTGGGGTAGTCTAAATCCCC**CTATAGTGAGTCGTATTAATTTC** |  |  |  |  |  |  |  |  |  |
| 1516-3 |  | *TTCTCTTTAACCGCCGATCGTATCGATG*GTTTTAGTCCCCTTCGTTTTTGGGGTAGTCTAAATCCCC**CTATAGTGAGTCGTATTAATTTC** |  |  |  |  |  |  |  |  |  |
| 1516-4 |  | *TCTCTTTAACCGCCGATCGTATCGATGA*GTTTTAGTCCCCTTCGTTTTTGGGGTAGTCTAAATCCCC**CTATAGTGAGTCGTATTAATTTC** |  |  |  |  |  |  |  |  |  |
| 1516-5 |  | *CTCTTTAACCGCCGATCGTATCGATGAT*GTTTTAGTCCCCTTCGTTTTTGGGGTAGTCTAAATCCCC**CTATAGTGAGTCGTATTAATTTC** |  |  |  |  |  |  |  |  |  |
| 1516-6 |  | *TCTTTAACCGCCGATCGTATCGATGATG*GTTTTAGTCCCCTTCGTTTTTGGGGTAGTCTAAATCCCC**CTATAGTGAGTCGTATTAATTTC** |  |  |  |  |  |  |  |  |  |
| 1516-7 |  | *CTTTAACCGCCGATCGTATCGATGATGA*GTTTTAGTCCCCTTCGTTTTTGGGGTAGTCTAAATCCCC**CTATAGTGAGTCGTATTAATTTC** |  |  |  |  |  |  |  |  |  |
| 1516-8 |  | *TTTAACCGCCGATCGTATCGATGATGAT*GTTTTAGTCCCCTTCGTTTTTGGGGTAGTCTAAATCCCC**CTATAGTGAGTCGTATTAATTTC** |  |  |  |  |  |  |  |  |  |
| 1516-9 |  | *TTAACCGCCGATCGTATCGATGATGATA*GTTTTAGTCCCCTTCGTTTTTGGGGTAGTCTAAATCCCC**CTATAGTGAGTCGTATTAATTTC** |  |  |  |  |  |  |  |  |  |
| 1516-10 |  | *TAACCGCCGATCGTATCGATGATGATAT*GTTTTAGTCCCCTTCGTTTTTGGGGTAGTCTAAATCCCC**CTATAGTGAGTCGTATTAATTTC** |  |  |  |  |  |  |  |  |  |
| 1516-11 |  | *AACCGCCGATCGTATCGATGATGATATT*GTTTTAGTCCCCTTCGTTTTTGGGGTAGTCTAAATCCCC**CTATAGTGAGTCGTATTAATTTC** |  |  |  |  |  |  |  |  |  |
| 1516-12 |  | *ACCGCCGATCGTATCGATGATGATATTT*GTTTTAGTCCCCTTCGTTTTTGGGGTAGTCTAAATCCCC**CTATAGTGAGTCGTATTAATTTC** |  |  |  |  |  |  |  |  |  |
| 1516-13 |  | *CCGCCGATCGTATCGATGATGATATTTT*GTTTTAGTCCCCTTCGTTTTTGGGGTAGTCTAAATCCCC**CTATAGTGAGTCGTATTAATTTC** |  |  |  |  |  |  |  |  |  |
| 1516-14 |  | *CGCCGATCGTATCGATGATGATATTTTG*GTTTTAGTCCCCTTCGTTTTTGGGGTAGTCTAAATCCCC**CTATAGTGAGTCGTATTAATTTC** |  |  |  |  |  |  |  |  |  |
| 1516-15 |  | *GCCGATCGTATCGATGATGATATTTTGA*GTTTTAGTCCCCTTCGTTTTTGGGGTAGTCTAAATCCCC**CTATAGTGAGTCGTATTAATTTC** |  |  |  |  |  |  |  |  |  |
| 1516-16 |  | *CCGATCGTATCGATGATGATATTTTGAA*GTTTTAGTCCCCTTCGTTTTTGGGGTAGTCTAAATCCCC**CTATAGTGAGTCGTATTAATTTC** |  |  |  |  |  |  |  |  |  |
| 1516-17 |  | *CGATCGTATCGATGATGATATTTTGAAA*GTTTTAGTCCCCTTCGTTTTTGGGGTAGTCTAAATCCCC**CTATAGTGAGTCGTATTAATTTC** |  |  |  |  |  |  |  |  |  |
| 1516-18 |  | *GATCGTATCGATGATGATATTTTGAAAG*GTTTTAGTCCCCTTCGTTTTTGGGGTAGTCTAAATCCCC**CTATAGTGAGTCGTATTAATTTC** |  |  |  |  |  |  |  |  |  |
| 1516-19 |  | *ATCGTATCGATGATGATATTTTGAAAGT*GTTTTAGTCCCCTTCGTTTTTGGGGTAGTCTAAATCCCC**CTATAGTGAGTCGTATTAATTTC** |  |  |  |  |  |  |  |  |  |
| 1516-20 |  | *TCGTATCGATGATGATATTTTGAAAGTG*GTTTTAGTCCCCTTCGTTTTTGGGGTAGTCTAAATCCCC**CTATAGTGAGTCGTATTAATTTC** |  |  |  |  |  |  |  |  |  |
| 1516-21 |  | *CGTATCGATGATGATATTTTGAAAGTGA*GTTTTAGTCCCCTTCGTTTTTGGGGTAGTCTAAATCCCC**CTATAGTGAGTCGTATTAATTTC** |  |  |  |  |  |  |  |  |  |
| 1516-22 |  | *GTATCGATGATGATATTTTGAAAGTGAT*GTTTTAGTCCCCTTCGTTTTTGGGGTAGTCTAAATCCCC**CTATAGTGAGTCGTATTAATTTC** |  |  |  |  |  |  |  |  |  |
| 1516-23 |  | *TATCGATGATGATATTTTGAAAGTGATT*GTTTTAGTCCCCTTCGTTTTTGGGGTAGTCTAAATCCCC**CTATAGTGAGTCGTATTAATTTC** |  |  |  |  |  |  |  |  |  |
| 1516-24 |  | *ATCGATGATGATATTTTGAAAGTGATTG*GTTTTAGTCCCCTTCGTTTTTGGGGTAGTCTAAATCCCC**CTATAGTGAGTCGTATTAATTTC** |  |  |  |  |  |  |  |  |  |
|  | crRNA15 | *TCGATGATGATATTTTGAAAGTGATTGT*GTTTTAGTCCCCTTCGTTTTTGGGGTAGTCTAAATCCCC**CTATAGTGAGTCGTATTAATTTC** |  |  |  |  |  |  |  |  |  |
| 1516-25 |  | *CGATGATGATATTTTGAAAGTGATTGTT*GTTTTAGTCCCCTTCGTTTTTGGGGTAGTCTAAATCCCC**CTATAGTGAGTCGTATTAATTTC** |  |  |  |  |  |  |  |  |  |
| 1516-26 |  | *GATGATGATATTTTGAAAGTGATTGTTG*GTTTTAGTCCCCTTCGTTTTTGGGGTAGTCTAAATCCCC**CTATAGTGAGTCGTATTAATTTC** |  |  |  |  |  |  |  |  |  |
| 1516-27 |  | *ATGATGATATTTTGAAAGTGATTGTTGA*GTTTTAGTCCCCTTCGTTTTTGGGGTAGTCTAAATCCCC**CTATAGTGAGTCGTATTAATTTC** |  |  |  |  |  |  |  |  |  |
| 1516-28 |  | *TGATGATATTTTGAAAGTGATTGTTGAT*GTTTTAGTCCCCTTCGTTTTTGGGGTAGTCTAAATCCCC**CTATAGTGAGTCGTATTAATTTC** |  |  |  |  |  |  |  |  |  |
| 1516-29 |  | *GATGATATTTTGAAAGTGATTGTTGATT*GTTTTAGTCCCCTTCGTTTTTGGGGTAGTCTAAATCCCC**CTATAGTGAGTCGTATTAATTTC** |  |  |  |  |  |  |  |  |  |
| 1516-30 |  | *ATGATATTTTGAAAGTGATTGTTGATTC*GTTTTAGTCCCCTTCGTTTTTGGGGTAGTCTAAATCCCC**CTATAGTGAGTCGTATTAATTTC** |  |  |  |  |  |  |  |  |  |
| 1516-31 |  | *TGATATTTTGAAAGTGATTGTTGATTCA*GTTTTAGTCCCCTTCGTTTTTGGGGTAGTCTAAATCCCC**CTATAGTGAGTCGTATTAATTTC** |  |  |  |  |  |  |  |  |  |
| 1516-32 |  | *GATATTTTGAAAGTGATTGTTGATTCAA*GTTTTAGTCCCCTTCGTTTTTGGGGTAGTCTAAATCCCC**CTATAGTGAGTCGTATTAATTTC** |  |  |  |  |  |  |  |  |  |
| 1516-33 |  | *ATATTTTGAAAGTGATTGTTGATTCAAT*GTTTTAGTCCCCTTCGTTTTTGGGGTAGTCTAAATCCCC**CTATAGTGAGTCGTATTAATTTC** |  |  |  |  |  |  |  |  |  |
| 1516-34 |  | *TATTTTGAAAGTGATTGTTGATTCAATG*GTTTTAGTCCCCTTCGTTTTTGGGGTAGTCTAAATCCCC**CTATAGTGAGTCGTATTAATTTC** |  |  |  |  |  |  |  |  |  |
| 1516-35 |  | *ATTTTGAAAGTGATTGTTGATTCAATGA*GTTTTAGTCCCCTTCGTTTTTGGGGTAGTCTAAATCCCC**CTATAGTGAGTCGTATTAATTTC** |  |  |  |  |  |  |  |  |  |
| 1516-36 |  | *TTTTGAAAGTGATTGTTGATTCAATGAA*GTTTTAGTCCCCTTCGTTTTTGGGGTAGTCTAAATCCCC**CTATAGTGAGTCGTATTAATTTC** |  |  |  |  |  |  |  |  |  |
| 1516-37 |  | *TTTGAAAGTGATTGTTGATTCAATGAAT*GTTTTAGTCCCCTTCGTTTTTGGGGTAGTCTAAATCCCC**CTATAGTGAGTCGTATTAATTTC** |  |  |  |  |  |  |  |  |  |
| 1516-38 |  | *TTGAAAGTGATTGTTGATTCAATGAATC*GTTTTAGTCCCCTTCGTTTTTGGGGTAGTCTAAATCCCC**CTATAGTGAGTCGTATTAATTTC** |  |  |  |  |  |  |  |  |  |
| 1516-39 |  | *TGAAAGTGATTGTTGATTCAATGAATCA*GTTTTAGTCCCCTTCGTTTTTGGGGTAGTCTAAATCCCC**CTATAGTGAGTCGTATTAATTTC** |  |  |  |  |  |  |  |  |  |
| 1516-40 |  | *GAAAGTGATTGTTGATTCAATGAATCAT*GTTTTAGTCCCCTTCGTTTTTGGGGTAGTCTAAATCCCC**CTATAGTGAGTCGTATTAATTTC** |  |  |  |  |  |  |  |  |  |
| 1516-41 |  | *AAAGTGATTGTTGATTCAATGAATCATC*GTTTTAGTCCCCTTCGTTTTTGGGGTAGTCTAAATCCCC**CTATAGTGAGTCGTATTAATTTC** |  |  |  |  |  |  |  |  |  |
| 1516-42 |  | *AAGTGATTGTTGATTCAATGAATCATCA*GTTTTAGTCCCCTTCGTTTTTGGGGTAGTCTAAATCCCC**CTATAGTGAGTCGTATTAATTTC** |  |  |  |  |  |  |  |  |  |
| 1516-43 |  | *AGTGATTGTTGATTCAATGAATCATCAT*GTTTTAGTCCCCTTCGTTTTTGGGGTAGTCTAAATCCCC**CTATAGTGAGTCGTATTAATTTC** |  |  |  |  |  |  |  |  |  |
| 1516-44 |  | *GTGATTGTTGATTCAATGAATCATCATG*GTTTTAGTCCCCTTCGTTTTTGGGGTAGTCTAAATCCCC**CTATAGTGAGTCGTATTAATTTC** |  |  |  |  |  |  |  |  |  |
| 1516-45 |  | *TGATTGTTGATTCAATGAATCATCATGG*GTTTTAGTCCCCTTCGTTTTTGGGGTAGTCTAAATCCCC**CTATAGTGAGTCGTATTAATTTC** |  |  |  |  |  |  |  |  |  |
| 1516-46 |  | *GATTGTTGATTCAATGAATCATCATGGT*GTTTTAGTCCCCTTCGTTTTTGGGGTAGTCTAAATCCCC**CTATAGTGAGTCGTATTAATTTC** |  |  |  |  |  |  |  |  |  |
| 1516-47 |  | *ATTGTTGATTCAATGAATCATCATGGTG*GTTTTAGTCCCCTTCGTTTTTGGGGTAGTCTAAATCCCC**CTATAGTGAGTCGTATTAATTTC** |  |  |  |  |  |  |  |  |  |
| 1516-48 |  | *TTGTTGATTCAATGAATCATCATGGTGA*GTTTTAGTCCCCTTCGTTTTTGGGGTAGTCTAAATCCCC**CTATAGTGAGTCGTATTAATTTC** |  |  |  |  |  |  |  |  |  |
| 1516-49 |  | *TGTTGATTCAATGAATCATCATGGTGAT*GTTTTAGTCCCCTTCGTTTTTGGGGTAGTCTAAATCCCC**CTATAGTGAGTCGTATTAATTTC** |  |  |  |  |  |  |  |  |  |
| 1516-50 |  | *GTTGATTCAATGAATCATCATGGTGATG*GTTTTAGTCCCCTTCGTTTTTGGGGTAGTCTAAATCCCC**CTATAGTGAGTCGTATTAATTTC** |  |  |  |  |  |  |  |  |  |
| 1516-51 |  | *TTGATTCAATGAATCATCATGGTGATGC*GTTTTAGTCCCCTTCGTTTTTGGGGTAGTCTAAATCCCC**CTATAGTGAGTCGTATTAATTTC** |  |  |  |  |  |  |  |  |  |
|  | crRNA16 | *TGATTCAATGAATCATCATGGTGATGCC*GTTTTAGTCCCCTTCGTTTTTGGGGTAGTCTAAATCCCC**CTATAGTGAGTCGTATTAATTTC** |  |  |  |  |  |  |  |  |  |
| 1516-52 |  | *GATTCAATGAATCATCATGGTGATGCCC*GTTTTAGTCCCCTTCGTTTTTGGGGTAGTCTAAATCCCC**CTATAGTGAGTCGTATTAATTTC** |  |  |  |  |  |  |  |  |  |
| 1516-53 |  | *ATTCAATGAATCATCATGGTGATGCCCG*GTTTTAGTCCCCTTCGTTTTTGGGGTAGTCTAAATCCCC**CTATAGTGAGTCGTATTAATTTC** |  |  |  |  |  |  |  |  |  |
| 1516-54 |  | *TTCAATGAATCATCATGGTGATGCCCGT*GTTTTAGTCCCCTTCGTTTTTGGGGTAGTCTAAATCCCC**CTATAGTGAGTCGTATTAATTTC** |  |  |  |  |  |  |  |  |  |
| 1516-55 |  | *TCAATGAATCATCATGGTGATGCCCGTA*GTTTTAGTCCCCTTCGTTTTTGGGGTAGTCTAAATCCCC**CTATAGTGAGTCGTATTAATTTC** |  |  |  |  |  |  |  |  |  |
| 1516-56 |  | *CAATGAATCATCATGGTGATGCCCGTAG*GTTTTAGTCCCCTTCGTTTTTGGGGTAGTCTAAATCCCC**CTATAGTGAGTCGTATTAATTTC** |  |  |  |  |  |  |  |  |  |
| 1516-57 |  | *AATGAATCATCATGGTGATGCCCGTAGC*GTTTTAGTCCCCTTCGTTTTTGGGGTAGTCTAAATCCCC**CTATAGTGAGTCGTATTAATTTC** |  |  |  |  |  |  |  |  |  |
| 1516-58 |  | *ATGAATCATCATGGTGATGCCCGTAGCA*GTTTTAGTCCCCTTCGTTTTTGGGGTAGTCTAAATCCCC**CTATAGTGAGTCGTATTAATTTC** |  |  |  |  |  |  |  |  |  |
| 1516-59 |  | *TGAATCATCATGGTGATGCCCGTAGCAA*GTTTTAGTCCCCTTCGTTTTTGGGGTAGTCTAAATCCCC**CTATAGTGAGTCGTATTAATTTC** |  |  |  |  |  |  |  |  |  |
| 1516-60 |  | *GAATCATCATGGTGATGCCCGTAGCAAG*GTTTTAGTCCCCTTCGTTTTTGGGGTAGTCTAAATCCCC**CTATAGTGAGTCGTATTAATTTC** |  |  |  |  |  |  |  |  |  |
| 1516-61 |  | *AATCATCATGGTGATGCCCGTAGCAAGT*GTTTTAGTCCCCTTCGTTTTTGGGGTAGTCTAAATCCCC**CTATAGTGAGTCGTATTAATTTC** |  |  |  |  |  |  |  |  |  |
| 1516-62 |  | *ATCATCATGGTGATGCCCGTAGCAAGTT*GTTTTAGTCCCCTTCGTTTTTGGGGTAGTCTAAATCCCC**CTATAGTGAGTCGTATTAATTTC** |  |  |  |  |  |  |  |  |  |
| 1516-63 |  | *TCATCATGGTGATGCCCGTAGCAAGTTG*GTTTTAGTCCCCTTCGTTTTTGGGGTAGTCTAAATCCCC**CTATAGTGAGTCGTATTAATTTC** |  |  |  |  |  |  |  |  |  |
| 1516-64 |  | *CATCATGGTGATGCCCGTAGCAAGTTGC*GTTTTAGTCCCCTTCGTTTTTGGGGTAGTCTAAATCCCC**CTATAGTGAGTCGTATTAATTTC** |  |  |  |  |  |  |  |  |  |
| 1516-65 |  | *ATCATGGTGATGCCCGTAGCAAGTTGCG*GTTTTAGTCCCCTTCGTTTTTGGGGTAGTCTAAATCCCC**CTATAGTGAGTCGTATTAATTTC** |  |  |  |  |  |  |  |  |  |
| 1516-66 |  | *TCATGGTGATGCCCGTAGCAAGTTGCGT*GTTTTAGTCCCCTTCGTTTTTGGGGTAGTCTAAATCCCC**CTATAGTGAGTCGTATTAATTTC** |  |  |  |  |  |  |  |  |  |
| 1516-67 |  | *CATGGTGATGCCCGTAGCAAGTTGCGTG*GTTTTAGTCCCCTTCGTTTTTGGGGTAGTCTAAATCCCC**CTATAGTGAGTCGTATTAATTTC** |  |  |  |  |  |  |  |  |  |
| 1516-68 |  | *ATGGTGATGCCCGTAGCAAGTTGCGTGA*GTTTTAGTCCCCTTCGTTTTTGGGGTAGTCTAAATCCCC**CTATAGTGAGTCGTATTAATTTC** |  |  |  |  |  |  |  |  |  |
| 1516-69 |  | *TGGTGATGCCCGTAGCAAGTTGCGTGAA*GTTTTAGTCCCCTTCGTTTTTGGGGTAGTCTAAATCCCC**CTATAGTGAGTCGTATTAATTTC** |  |  |  |  |  |  |  |  |  |
| 1516-70 |  | *GGTGATGCCCGTAGCAAGTTGCGTGAAG*GTTTTAGTCCCCTTCGTTTTTGGGGTAGTCTAAATCCCC**CTATAGTGAGTCGTATTAATTTC** |  |  |  |  |  |  |  |  |  |
| 1516-71 |  | *GTGATGCCCGTAGCAAGTTGCGTGAAGA*GTTTTAGTCCCCTTCGTTTTTGGGGTAGTCTAAATCCCC**CTATAGTGAGTCGTATTAATTTC** |  |  |  |  |  |  |  |  |  |
| 1516-72 |  | *TGATGCCCGTAGCAAGTTGCGTGAAGAA*GTTTTAGTCCCCTTCGTTTTTGGGGTAGTCTAAATCCCC**CTATAGTGAGTCGTATTAATTTC** |  |  |  |  |  |  |  |  |  |
| 1516-73 |  | *GATGCCCGTAGCAAGTTGCGTGAAGAAT*GTTTTAGTCCCCTTCGTTTTTGGGGTAGTCTAAATCCCC**CTATAGTGAGTCGTATTAATTTC** |  |  |  |  |  |  |  |  |  |
| 1516-74 |  | *ATGCCCGTAGCAAGTTGCGTGAAGAATT*GTTTTAGTCCCCTTCGTTTTTGGGGTAGTCTAAATCCCC**CTATAGTGAGTCGTATTAATTTC** |  |  |  |  |  |  |  |  |  |
| 2021-1 |  | *TTCAAGCCGAAATTAATAAGCATCTGTC*GTTTTAGTCCCCTTCGTTTTTGGGGTAGTCTAAATCCCC**CTATAGTGAGTCGTATTAATTTC** |  |  |  |  |  |  |  |  |  |
| 2021-2 |  | *TCAAGCCGAAATTAATAAGCATCTGTCT*GTTTTAGTCCCCTTCGTTTTTGGGGTAGTCTAAATCCCC**CTATAGTGAGTCGTATTAATTTC** |  |  |  |  |  |  |  |  |  |
| 2021-3 |  | *CAAGCCGAAATTAATAAGCATCTGTCTA*GTTTTAGTCCCCTTCGTTTTTGGGGTAGTCTAAATCCCC**CTATAGTGAGTCGTATTAATTTC** |  |  |  |  |  |  |  |  |  |
| 2021-4 |  | *AAGCCGAAATTAATAAGCATCTGTCTAG*GTTTTAGTCCCCTTCGTTTTTGGGGTAGTCTAAATCCCC**CTATAGTGAGTCGTATTAATTTC** |  |  |  |  |  |  |  |  |  |
| 2021-5 |  | *AGCCGAAATTAATAAGCATCTGTCTAGT*GTTTTAGTCCCCTTCGTTTTTGGGGTAGTCTAAATCCCC**CTATAGTGAGTCGTATTAATTTC** |  |  |  |  |  |  |  |  |  |
| 2021-6 |  | *GCCGAAATTAATAAGCATCTGTCTAGTA*GTTTTAGTCCCCTTCGTTTTTGGGGTAGTCTAAATCCCC**CTATAGTGAGTCGTATTAATTTC** |  |  |  |  |  |  |  |  |  |
| 2021-7 |  | *CCGAAATTAATAAGCATCTGTCTAGTAG*GTTTTAGTCCCCTTCGTTTTTGGGGTAGTCTAAATCCCC**CTATAGTGAGTCGTATTAATTTC** |  |  |  |  |  |  |  |  |  |
| 2021-8 |  | *CGAAATTAATAAGCATCTGTCTAGTAGT*GTTTTAGTCCCCTTCGTTTTTGGGGTAGTCTAAATCCCC**CTATAGTGAGTCGTATTAATTTC** |  |  |  |  |  |  |  |  |  |
| 2021-9 |  | *GAAATTAATAAGCATCTGTCTAGTAGTG*GTTTTAGTCCCCTTCGTTTTTGGGGTAGTCTAAATCCCC**CTATAGTGAGTCGTATTAATTTC** |  |  |  |  |  |  |  |  |  |
| 2021-10 |  | *AAATTAATAAGCATCTGTCTAGTAGTGG*GTTTTAGTCCCCTTCGTTTTTGGGGTAGTCTAAATCCCC**CTATAGTGAGTCGTATTAATTTC** |  |  |  |  |  |  |  |  |  |
| 2021-11 |  | *AATTAATAAGCATCTGTCTAGTAGTGGC*GTTTTAGTCCCCTTCGTTTTTGGGGTAGTCTAAATCCCC**CTATAGTGAGTCGTATTAATTTC** |  |  |  |  |  |  |  |  |  |
| 2021-12 |  | *ATTAATAAGCATCTGTCTAGTAGTGGCA*GTTTTAGTCCCCTTCGTTTTTGGGGTAGTCTAAATCCCC**CTATAGTGAGTCGTATTAATTTC** |  |  |  |  |  |  |  |  |  |
| 2021-13 |  | *TTAATAAGCATCTGTCTAGTAGTGGCAC*GTTTTAGTCCCCTTCGTTTTTGGGGTAGTCTAAATCCCC**CTATAGTGAGTCGTATTAATTTC** |  |  |  |  |  |  |  |  |  |
| 2021-14 |  | *TAATAAGCATCTGTCTAGTAGTGGCACC*GTTTTAGTCCCCTTCGTTTTTGGGGTAGTCTAAATCCCC**CTATAGTGAGTCGTATTAATTTC** |  |  |  |  |  |  |  |  |  |
| 2021-15 |  | *AATAAGCATCTGTCTAGTAGTGGCACCA*GTTTTAGTCCCCTTCGTTTTTGGGGTAGTCTAAATCCCC**CTATAGTGAGTCGTATTAATTTC** |  |  |  |  |  |  |  |  |  |
| 2021-16 |  | *ATAAGCATCTGTCTAGTAGTGGCACCAT*GTTTTAGTCCCCTTCGTTTTTGGGGTAGTCTAAATCCCC**CTATAGTGAGTCGTATTAATTTC** |  |  |  |  |  |  |  |  |  |
| 2021-17 |  | *TAAGCATCTGTCTAGTAGTGGCACCATA*GTTTTAGTCCCCTTCGTTTTTGGGGTAGTCTAAATCCCC**CTATAGTGAGTCGTATTAATTTC** |  |  |  |  |  |  |  |  |  |
| 2021-18 |  | *AAGCATCTGTCTAGTAGTGGCACCATAA*GTTTTAGTCCCCTTCGTTTTTGGGGTAGTCTAAATCCCC**CTATAGTGAGTCGTATTAATTTC** |  |  |  |  |  |  |  |  |  |
| 2021-19 |  | *AGCATCTGTCTAGTAGTGGCACCATAAA*GTTTTAGTCCCCTTCGTTTTTGGGGTAGTCTAAATCCCC**CTATAGTGAGTCGTATTAATTTC** |  |  |  |  |  |  |  |  |  |
| 2021-20 |  | *GCATCTGTCTAGTAGTGGCACCATAAAT*GTTTTAGTCCCCTTCGTTTTTGGGGTAGTCTAAATCCCC**CTATAGTGAGTCGTATTAATTTC** |  |  |  |  |  |  |  |  |  |
| 2021-21 |  | *CATCTGTCTAGTAGTGGCACCATAAATA*GTTTTAGTCCCCTTCGTTTTTGGGGTAGTCTAAATCCCC**CTATAGTGAGTCGTATTAATTTC** |  |  |  |  |  |  |  |  |  |
| 2021-22 |  | *ATCTGTCTAGTAGTGGCACCATAAATAT*GTTTTAGTCCCCTTCGTTTTTGGGGTAGTCTAAATCCCC**CTATAGTGAGTCGTATTAATTTC** |  |  |  |  |  |  |  |  |  |
| 2021-23 |  | *TCTGTCTAGTAGTGGCACCATAAATATC*GTTTTAGTCCCCTTCGTTTTTGGGGTAGTCTAAATCCCC**CTATAGTGAGTCGTATTAATTTC** |  |  |  |  |  |  |  |  |  |
| 2021-24 |  | *CTGTCTAGTAGTGGCACCATAAATATCC*GTTTTAGTCCCCTTCGTTTTTGGGGTAGTCTAAATCCCC**CTATAGTGAGTCGTATTAATTTC** |  |  |  |  |  |  |  |  |  |
|  | crRNA20 | *TGTCTAGTAGTGGCACCATAAATATCCA*GTTTTAGTCCCCTTCGTTTTTGGGGTAGTCTAAATCCCC**CTATAGTGAGTCGTATTAATTTC** |  |  |  |  |  |  |  |  |  |
| 2021-25 |  | *GTCTAGTAGTGGCACCATAAATATCCAT*GTTTTAGTCCCCTTCGTTTTTGGGGTAGTCTAAATCCCC**CTATAGTGAGTCGTATTAATTTC** |  |  |  |  |  |  |  |  |  |
| 2021-26 |  | *TCTAGTAGTGGCACCATAAATATCCATG*GTTTTAGTCCCCTTCGTTTTTGGGGTAGTCTAAATCCCC**CTATAGTGAGTCGTATTAATTTC** |  |  |  |  |  |  |  |  |  |
| 2021-27 |  | *CTAGTAGTGGCACCATAAATATCCATGA*GTTTTAGTCCCCTTCGTTTTTGGGGTAGTCTAAATCCCC**CTATAGTGAGTCGTATTAATTTC** |  |  |  |  |  |  |  |  |  |
| 2021-28 |  | *TAGTAGTGGCACCATAAATATCCATGAT*GTTTTAGTCCCCTTCGTTTTTGGGGTAGTCTAAATCCCC**CTATAGTGAGTCGTATTAATTTC** |  |  |  |  |  |  |  |  |  |
| 2021-29 |  | *AGTAGTGGCACCATAAATATCCATGATA*GTTTTAGTCCCCTTCGTTTTTGGGGTAGTCTAAATCCCC**CTATAGTGAGTCGTATTAATTTC** |  |  |  |  |  |  |  |  |  |
| 2021-30 |  | *GTAGTGGCACCATAAATATCCATGATAA*GTTTTAGTCCCCTTCGTTTTTGGGGTAGTCTAAATCCCC**CTATAGTGAGTCGTATTAATTTC** |  |  |  |  |  |  |  |  |  |
| 2021-31 |  | *TAGTGGCACCATAAATATCCATGATAAA*GTTTTAGTCCCCTTCGTTTTTGGGGTAGTCTAAATCCCC**CTATAGTGAGTCGTATTAATTTC** |  |  |  |  |  |  |  |  |  |
| 2021-32 |  | *AGTGGCACCATAAATATCCATGATAAAT*GTTTTAGTCCCCTTCGTTTTTGGGGTAGTCTAAATCCCC**CTATAGTGAGTCGTATTAATTTC** |  |  |  |  |  |  |  |  |  |
| 2021-33 |  | *GTGGCACCATAAATATCCATGATAAATC*GTTTTAGTCCCCTTCGTTTTTGGGGTAGTCTAAATCCCC**CTATAGTGAGTCGTATTAATTTC** |  |  |  |  |  |  |  |  |  |
| 2021-34 |  | *TGGCACCATAAATATCCATGATAAATCC*GTTTTAGTCCCCTTCGTTTTTGGGGTAGTCTAAATCCCC**CTATAGTGAGTCGTATTAATTTC** |  |  |  |  |  |  |  |  |  |
| 2021-35 |  | *GGCACCATAAATATCCATGATAAATCCA*GTTTTAGTCCCCTTCGTTTTTGGGGTAGTCTAAATCCCC**CTATAGTGAGTCGTATTAATTTC** |  |  |  |  |  |  |  |  |  |
| 2021-36 |  | *GCACCATAAATATCCATGATAAATCCAT*GTTTTAGTCCCCTTCGTTTTTGGGGTAGTCTAAATCCCC**CTATAGTGAGTCGTATTAATTTC** |  |  |  |  |  |  |  |  |  |
| 2021-37 |  | *CACCATAAATATCCATGATAAATCCATT*GTTTTAGTCCCCTTCGTTTTTGGGGTAGTCTAAATCCCC**CTATAGTGAGTCGTATTAATTTC** |  |  |  |  |  |  |  |  |  |
| 2021-38 |  | *ACCATAAATATCCATGATAAATCCATTA*GTTTTAGTCCCCTTCGTTTTTGGGGTAGTCTAAATCCCC**CTATAGTGAGTCGTATTAATTTC** |  |  |  |  |  |  |  |  |  |
| 2021-39 |  | *CCATAAATATCCATGATAAATCCATTAA*GTTTTAGTCCCCTTCGTTTTTGGGGTAGTCTAAATCCCC**CTATAGTGAGTCGTATTAATTTC** |  |  |  |  |  |  |  |  |  |
| 2021-40 |  | *CATAAATATCCATGATAAATCCATTAAT*GTTTTAGTCCCCTTCGTTTTTGGGGTAGTCTAAATCCCC**CTATAGTGAGTCGTATTAATTTC** |  |  |  |  |  |  |  |  |  |
| 2021-41 |  | *ATAAATATCCATGATAAATCCATTAATC*GTTTTAGTCCCCTTCGTTTTTGGGGTAGTCTAAATCCCC**CTATAGTGAGTCGTATTAATTTC** |  |  |  |  |  |  |  |  |  |
| 2021-42 |  | *TAAATATCCATGATAAATCCATTAATCT*GTTTTAGTCCCCTTCGTTTTTGGGGTAGTCTAAATCCCC**CTATAGTGAGTCGTATTAATTTC** |  |  |  |  |  |  |  |  |  |
| 2021-43 |  | *AAATATCCATGATAAATCCATTAATCTC*GTTTTAGTCCCCTTCGTTTTTGGGGTAGTCTAAATCCCC**CTATAGTGAGTCGTATTAATTTC** |  |  |  |  |  |  |  |  |  |
| 2021-44 |  | *AATATCCATGATAAATCCATTAATCTCA*GTTTTAGTCCCCTTCGTTTTTGGGGTAGTCTAAATCCCC**CTATAGTGAGTCGTATTAATTTC** |  |  |  |  |  |  |  |  |  |
| 2021-45 |  | *ATATCCATGATAAATCCATTAATCTCAT*GTTTTAGTCCCCTTCGTTTTTGGGGTAGTCTAAATCCCC**CTATAGTGAGTCGTATTAATTTC** |  |  |  |  |  |  |  |  |  |
| 2021-46 |  | *TATCCATGATAAATCCATTAATCTCATG*GTTTTAGTCCCCTTCGTTTTTGGGGTAGTCTAAATCCCC**CTATAGTGAGTCGTATTAATTTC** |  |  |  |  |  |  |  |  |  |
| 2021-47 |  | *ATCCATGATAAATCCATTAATCTCATGG*GTTTTAGTCCCCTTCGTTTTTGGGGTAGTCTAAATCCCC**CTATAGTGAGTCGTATTAATTTC** |  |  |  |  |  |  |  |  |  |
| 2021-48 |  | *TCCATGATAAATCCATTAATCTCATGGA*GTTTTAGTCCCCTTCGTTTTTGGGGTAGTCTAAATCCCC**CTATAGTGAGTCGTATTAATTTC** |  |  |  |  |  |  |  |  |  |
| 2021-49 |  | *CCATGATAAATCCATTAATCTCATGGAT*GTTTTAGTCCCCTTCGTTTTTGGGGTAGTCTAAATCCCC**CTATAGTGAGTCGTATTAATTTC** |  |  |  |  |  |  |  |  |  |
| 2021-50 |  | *CATGATAAATCCATTAATCTCATGGATA*GTTTTAGTCCCCTTCGTTTTTGGGGTAGTCTAAATCCCC**CTATAGTGAGTCGTATTAATTTC** |  |  |  |  |  |  |  |  |  |
| 2021-51 | crRNA21 | *ATGATAAATCCATTAATCTCATGGATAA*GTTTTAGTCCCCTTCGTTTTTGGGGTAGTCTAAATCCCC**CTATAGTGAGTCGTATTAATTTC** |  |  |  |  |  |  |  |  |  |
| 2021-52 |  | *GATAAATCCATTAATCTCATGGATAAAA*GTTTTAGTCCCCTTCGTTTTTGGGGTAGTCTAAATCCCC**CTATAGTGAGTCGTATTAATTTC** |  |  |  |  |  |  |  |  |  |
| 2021-53 |  | *ATAAATCCATTAATCTCATGGATAAAAA*GTTTTAGTCCCCTTCGTTTTTGGGGTAGTCTAAATCCCC**CTATAGTGAGTCGTATTAATTTC** |  |  |  |  |  |  |  |  |  |
| 2021-54 |  | *TAAATCCATTAATCTCATGGATAAAAAT*GTTTTAGTCCCCTTCGTTTTTGGGGTAGTCTAAATCCCC**CTATAGTGAGTCGTATTAATTTC** |  |  |  |  |  |  |  |  |  |
| 2021-55 |  | *AAATCCATTAATCTCATGGATAAAAATT*GTTTTAGTCCCCTTCGTTTTTGGGGTAGTCTAAATCCCC**CTATAGTGAGTCGTATTAATTTC** |  |  |  |  |  |  |  |  |  |
| 2021-56 |  | *AATCCATTAATCTCATGGATAAAAATTT*GTTTTAGTCCCCTTCGTTTTTGGGGTAGTCTAAATCCCC**CTATAGTGAGTCGTATTAATTTC** |  |  |  |  |  |  |  |  |  |
| 2021-57 |  | *ATCCATTAATCTCATGGATAAAAATTTA*GTTTTAGTCCCCTTCGTTTTTGGGGTAGTCTAAATCCCC**CTATAGTGAGTCGTATTAATTTC** |  |  |  |  |  |  |  |  |  |
| 2021-58 |  | *TCCATTAATCTCATGGATAAAAATTTAT*GTTTTAGTCCCCTTCGTTTTTGGGGTAGTCTAAATCCCC**CTATAGTGAGTCGTATTAATTTC** |  |  |  |  |  |  |  |  |  |
| 2021-59 |  | *CCATTAATCTCATGGATAAAAATTTATA*GTTTTAGTCCCCTTCGTTTTTGGGGTAGTCTAAATCCCC**CTATAGTGAGTCGTATTAATTTC** |  |  |  |  |  |  |  |  |  |
| 2021-60 |  | *CATTAATCTCATGGATAAAAATTTATAT*GTTTTAGTCCCCTTCGTTTTTGGGGTAGTCTAAATCCCC**CTATAGTGAGTCGTATTAATTTC** |  |  |  |  |  |  |  |  |  |
| 2021-61 |  | *ATTAATCTCATGGATAAAAATTTATATG*GTTTTAGTCCCCTTCGTTTTTGGGGTAGTCTAAATCCCC**CTATAGTGAGTCGTATTAATTTC** |  |  |  |  |  |  |  |  |  |
| 2021-62 |  | *TTAATCTCATGGATAAAAATTTATATGG*GTTTTAGTCCCCTTCGTTTTTGGGGTAGTCTAAATCCCC**CTATAGTGAGTCGTATTAATTTC** |  |  |  |  |  |  |  |  |  |
| 2021-63 |  | *TAATCTCATGGATAAAAATTTATATGGT*GTTTTAGTCCCCTTCGTTTTTGGGGTAGTCTAAATCCCC**CTATAGTGAGTCGTATTAATTTC** |  |  |  |  |  |  |  |  |  |
| 2021-64 |  | *AATCTCATGGATAAAAATTTATATGGTT*GTTTTAGTCCCCTTCGTTTTTGGGGTAGTCTAAATCCCC**CTATAGTGAGTCGTATTAATTTC** |  |  |  |  |  |  |  |  |  |
| 2021-65 |  | *ATCTCATGGATAAAAATTTATATGGTTA*GTTTTAGTCCCCTTCGTTTTTGGGGTAGTCTAAATCCCC**CTATAGTGAGTCGTATTAATTTC** |  |  |  |  |  |  |  |  |  |
| 2021-66 |  | *TCTCATGGATAAAAATTTATATGGTTAT*GTTTTAGTCCCCTTCGTTTTTGGGGTAGTCTAAATCCCC**CTATAGTGAGTCGTATTAATTTC** |  |  |  |  |  |  |  |  |  |
| 2021-67 |  | *CTCATGGATAAAAATTTATATGGTTATA*GTTTTAGTCCCCTTCGTTTTTGGGGTAGTCTAAATCCCC**CTATAGTGAGTCGTATTAATTTC** |  |  |  |  |  |  |  |  |  |
| 2021-68 |  | *TCATGGATAAAAATTTATATGGTTATAC*GTTTTAGTCCCCTTCGTTTTTGGGGTAGTCTAAATCCCC**CTATAGTGAGTCGTATTAATTTC** |  |  |  |  |  |  |  |  |  |
| 2021-69 |  | *CATGGATAAAAATTTATATGGTTATACA*GTTTTAGTCCCCTTCGTTTTTGGGGTAGTCTAAATCCCC**CTATAGTGAGTCGTATTAATTTC** |  |  |  |  |  |  |  |  |  |
| 2021-70 |  | *ATGGATAAAAATTTATATGGTTATACAG*GTTTTAGTCCCCTTCGTTTTTGGGGTAGTCTAAATCCCC**CTATAGTGAGTCGTATTAATTTC** |  |  |  |  |  |  |  |  |  |
| 2021-71 |  | *TGGATAAAAATTTATATGGTTATACAGA*GTTTTAGTCCCCTTCGTTTTTGGGGTAGTCTAAATCCCC**CTATAGTGAGTCGTATTAATTTC** |  |  |  |  |  |  |  |  |  |
| 2021-72 |  | *GGATAAAAATTTATATGGTTATACAGAT*GTTTTAGTCCCCTTCGTTTTTGGGGTAGTCTAAATCCCC**CTATAGTGAGTCGTATTAATTTC** |  |  |  |  |  |  |  |  |  |
| 2021-73 |  | *GATAAAAATTTATATGGTTATACAGATG*GTTTTAGTCCCCTTCGTTTTTGGGGTAGTCTAAATCCCC**CTATAGTGAGTCGTATTAATTTC** |  |  |  |  |  |  |  |  |  |
| 2021-74 |  | *ATAAAAATTTATATGGTTATACAGATGA*GTTTTAGTCCCCTTCGTTTTTGGGGTAGTCTAAATCCCC**CTATAGTGAGTCGTATTAATTTC** |  |  |  |  |  |  |  |  |  |

Supplemental Table 2. PCR Primers Used

|  |  |  |
| --- | --- | --- |
| **Primer Name** | **Sequence (5’-3’)** |  |
|  |  |  |
|  |  |  |
| T7lcrVF | GAAATTAATACGACTCACTATAGAATGATTAGAGCCTACGAACAAAACCC |  |
| YpestislcrVR | TCATTTACCAGACGTGTCATCTAGCA |  |
| RPA56F | GAAATTAATACGACTCACTATAGATTGAGGATCTAGAAAAAGTTAGGGTGGA |  |
| RPA56R | GTCATAATGACCGCCTTTAAGAATGGC |  |
|  |  |  |
| T7F | GAAATTAATACGACTCACTATAG |  |
